# Fungal-Based Biogenic Silver Nanoparticles: Synthesis, Mathematical Modeling and Application

**DOI:** 10.3390/molecules31173103

**Published:** 2026-09-04

**Authors:** Alejandra Ortiz-Tirado, Erick R. Bandala, Victor H. Robledo-Zacarias, Alain S. Conejo-Davila, Alejandro Vega-Rios, James McGree, Luis Fernando Mejia-Diaz, Ashantha Goonetilleke, Martín Pacheco-Álvarez, Oscar M. Rodriguez-Narvaez

**Affiliations:** 1 Direction of Applied Research and Development, Center for Applied Innovation in Competitive Technologies, Omega 201, León 37545, Guanajuato, Mexico; nortiz.picyt@ciatec.mx; 2 NantBioRenewables LLC, 100 Seapoint Blv. Bldg 5, Savannah, GA 31404, USA; erick.bandala@nantrenewables.com; 3 Technological Solutions Department, Center for Applied Innovation in Competitive Technologies, Omega 201, León 37545, Guanajuato, Mexico; vrobledo@ciatec.mx; 4 MicroPrime S RL de CV, El Colorado, Santiago de Querétaro 76246, Querétaro, Mexico; 5 Centro de Investigación en Materiales Avanzados, S.C. (CIMAV), Miguel de Cervantes No. 120, Chihuahua 31136, Chihuahua, Mexico; alejandro.vega@cimav.edu.mx; 6 School of Mathematical Sciences, Queensland University of Technology, GPO Box 2344, Brisbane, QLD 4001, Australia; james.mcgree@qut.edu.au; 7 Transdisciplinary Institute of Research and Services (ITRANS), University of Guadalajara, Zapopan 45150, Jalisco, Mexico; mejia.fer93@gmail.com; 8 Department of Civil Engineering, Birla Institute of Technology and Science, Pilani Campus, Vidya Vihar, Pilani 333031, Rajasthan, India; ashanthag@pm.me; 9 Departamento de Química-DCNE, Universidad de Guanajuato, Cerro de la Venada s/n, Pueblito de Rocha, Guanajuato 36040, Guanajuato, Mexico; moa.pachecoalvarez@ugto.mx; 10 Tecnológico Nacional de México, Instituto Tecnológico Superior de Guanajuato, Carretera Estatal Guanajuato-Puentecillas Km. 10.5, Guanajuato 36262, Guanajuato, Mexico

**Keywords:** biogenic synthesis, filamentous fungus, silver nanoparticles, disinfection, biomolecules

## Abstract

Biogenic synthesis based on microorganisms and plants utilizes biomolecules and metabolites that act as reducing and stabilizing agents. Furthermore, these biomolecules confer enhanced properties, increasing their effectiveness as antibacterial agents, even against multidrug-resistant bacteria. However, there is currently a lack of knowledge regarding the characteristics of the biomolecules involved and their mechanisms during synthesis, which limits their wider application. This research study focused on the synthesis of AgNPs using extracellularly secreted biomolecules from the filamentous fungus *R. stolonifer*. The results indicated that the concentration of the biomolecules depends on the response of the fungus to specific operational conditions. Furthermore, a relationship was found between the concentrations of total proteins and polyphenols, an optimal protein-to-polyphenol ratio, and the formation of AgNPs. Characterization of the fungus extracts revealed a complex mixture of over 100 metabolites, primarily phenolic compounds, proteins, and methoxylated compounds, thereby identifying a synergistic mechanism that enables the reduction and stabilization of AgNPs. Disinfection tests confirmed the antibacterial efficacy of all biosynthesized AgNPs, achieving 100% elimination of *Escherichia coli* and *Staphylococcus aureus*. The study outcomes highlight the important role of fungal biomolecules for creating a sustainable and efficient method for synthesizing antimicrobial nanoparticles with high efficacy for disinfectant applications.

## 1. Introduction

In recent years, the use of nanoparticles (NPs) in water treatment has emerged as an attractive alternative due to their physical and chemical properties, particularly those with antibacterial, antifungal, and antiviral capabilities [1,2,3], such as silver nanoparticles (AgNPs), which have demonstrated potent antimicrobial properties, including superior efficacy against pathogenic bacterial strains such as Escherichia coli and Staphylococcus aureus compared to other metal-based NPs [4,5,6,7]. Their use as a disinfectant is emerging as a promising alternative, offering a more sustainable, efficient, and environmentally friendly approach, particularly when AgNPs are synthesized using biogenic methods that employ microbially derived and plant-derived byproducts as reducing agents and stabilizers [4,8,9,10,11]. Biogenic synthesis based on unicellular and multicellular microorganisms (e.g., bacteria, fungi, yeasts, algae) from diverse environments, including soil, water, and plants [3,4,8], offers clear advantages due to the non-toxic nature and the enhanced properties conferred on the synthesized nanoparticles, such as biocompatibility and increased antibacterial and antifungal activity [1,2], including high efficacy against multidrug-resistant pathogenic bacteria [12,13]. The synthesis process using microorganisms involves the use of biomolecules and metabolites produced and secreted under specific conditions applied during their cultivation [10,14,15]. Common inducers rely on nutrient availability, temperature variation, or pH changes to stimulate the secretion of specific biomolecules [16]. The biomolecules available for NP formation depend on the inducer and the mechanism used: intracellular, where ions are reduced within microbial cells by enzymes, proteins, and other intracellular and extracellular biomolecules acting as mediators, or extracellular, which relies exclusively on metabolites secreted by the microorganism in the surrounding medium [1,9,17]. Extracellular pathways are often preferred due to their shorter processing times and simpler methodologies compared to intracellular routes, which require additional steps to extract nanoparticles from the cells [1,9]. Despite the advantages of microorganism-based synthesis, significant knowledge gaps remain regarding extraction methods for obtaining specific biomolecules, as these strongly influence nanoparticle physicochemical properties and functionality. Also, understanding the biomolecular species involved in NP is particularly important in microbial systems, where biological variability can affect reproducibility. Further, although biogenic synthesis offers environmental and economic advantages and opportunities for waste valorization, challenges remain regarding scalability, stability, biocompatibility, ecological impacts, and production costs. Further, integrating life-cycle assessment (LCA), computational modeling, and comprehensive management strategies is essential for establishing biogenic nanotechnology as a safe, sustainable, and scalable alternative [18,19]. However, these aspects are outside the scope of the current study and have not been investigated. This study explores the biogenic synthesis of AgNPs using extracts from the filamentous fungus *Rhizopus stolonifer*, evaluating different conditions and systematically characterizing the fungus-derived biomolecules present in a diverse range of fungal extracts, in addition to assessing the antibacterial capability of the biosynthesized AgNPs. The study outcomes are expected to provide knowledge to enhance the synthesis of more effective and sustainable disinfection products for water treatment, as the systematic understanding of the influence of extraction conditions and biomolecular composition can support the future optimization and scale-up of biogenic AgNP production.

## 2. Materials and Methods

### 2.1. Reagents

The reagents used in this study, including Potato dextrose agar (PDA, P2182 Millipore, Millipore, Burlington, MA, USA) solid medium, silver nitrate (AgNO_3_, Merck, Naucalpan de Juárez, Mexico), Bradford solution (Quick Start, Bio-Rad, Hercules, CA, USA), Folin’s reagent (2790 HYCEL, Zapopan, Mexico), sodium carbonate (Na_2_CO_3_, 3598 JT BAKER, Aguascalientes, Mexico), bovine serum albumin (BSA, A8806 Sigma, Sigma, Burbank, CA, USA), and gallic acid (Merck, Naucalpan de Juárez, Mexico), were used as received.

### 2.2. Fungus Extract Preparation

A fungus isolated from *Artocarpus heterophyllus* (Jackfruit), identified as *Rhizopus stolonifer*, was used to study biomass production kinetics using the dry weight method, in which fungal samples are collected on filter paper at different growth stages and dried at 100 °C for 24 h. The radial growth kinetics were monitored by measuring the increase in diameter every 24 h until the fungus reached the maximum diameter of the Petri dish. Both kinetics were performed using a circular pellet of known diameter and weight cut from a previous fungal culture using potato dextrose agar (PDA) as the solid medium and an incubation temperature of 28 ± 1 °C. The extract was prepared by mixing the biomass with sterile distilled water and keeping it at room temperature (28 ± 1 °C) with constant stirring at 120 rpm. After filtration through a 0.45 µm Millipore membrane, the supernatant was retained and stored at 4 °C.

### 2.3. Biogenic Synthesis of Silver Nanoparticles (AgNPs)

Silver nitrate (AgNO_3_) was added to the fungus extract to reach a final concentration of 1 mM and stirred at 120 rpm at 28 ± 1 °C for up to 24 h or until a visible orange color change occurred in a Thermo Scientific stirring plate with stirring and heating control (Mexico City, Mexico). The color change observed during the synthesis of silver nanoparticles (AgNPs) is a preliminary indicator of their formation. This phenomenon is because AgNPs present a coloration different from that of the metal on its macroscopic scale, resulting in the color change in the solution and the presence of surface plasmon resonance (SPR) characteristic of the formation of AgNPs. The solution was then stored at 4 °C two weeks before being completely used or characterized.

### 2.4. Characterization of the Fungus Extracts

The fungus extracts were characterized by Fourier Transform Infrared Spectroscopy (FT-IR) using a Thermo Scientific NICOLET IS10 instrument and by absorption scanning (200 to 700 nm) using a Thermo Scientific, evolution 300 UV-vis spectrophotometer (Thermo Scientific, Waltham, MA, USA). Colorimetric techniques were employed to quantify total proteins and polyphenols using methods adapted from Bradford [20,21] and Folin–Ciocalteu [22,23], respectively.

For the modified Bradford method, 10 µL of the sample was mixed with 200 µL of Bradford solution (i.e., 1:4 volume/volume rate). For the modified Folin–Ciocalteu method, 3 µL of the sample was mixed with 237 µL of distilled water, 15 µL of 10% Folin’s reagent, and 45 µL of 15% sodium carbonate (Na_2_CO_3_). A 96-well microplate was used for both methods. The reactions were allowed to proceed for five minutes in the dark, and the absorbance was measured using a UV-Vis spectrophotometer (Thermo Scientific, Waltham, MA, USA) at 595 nm for protein quantification and 760 nm for polyphenol quantification. Calibration curves for the protein and phenolic compounds were generated using bovine serum albumin (BSA) and gallic acid, respectively.

### 2.5. Screening of Fungus Extract by GC-MS Analysis

To profile the active components, the fungus extract was evaporated to dryness at 40 °C using a rotary vacuum evaporator under a nitrogen atmosphere. The resulting crude residue was resuspended in 1 mL of methanol and filtered through a 0.22 µm membrane. To ensure complete removal of water, the solution was dried again with anhydrous sodium sulfate.

A 2 µL sample was injected into an Agilent 7890 GC coupled to a 5975C MSD (Agilent Technologies, Santa Clara, CA, USA) with a 20:1 split ratio. Separation was performed using an HP-5ms Ultra Inert column (30 m × 0.25 mm, 0.25 µm; Agilent J&W). The flow rate of the helium carrier was maintained at 1.2 mL min^−1^. The column oven program was set at 35 °C, then the temperature was increased to 325 °C (5 °C min^−1^) and maintained till the end of the chromatographic run (60 min). The transfer line and ion source temperature were maintained at 300 °C and 230 °C, respectively. Electron ionization (EI) was performed at 70 eV. Chromatograms were acquired in full-scan mode over the *m*/*z* range of 29–550 using MassHunter Workstation Software (Software Version B.07.00/Build 7.0.457.0.). For identification, raw data were exported and processed using Agilent Unknowns analysis software (The NIST Mass Spectral Search Program for de NIST/EPA/NIH Mass Spectral Library Version 2.0 Buil May 19 2011), and compounds were identified by comparing mass spectra and retention times against the National Institute of Standards and Technology (NIST version 2.0) standard library database.

### 2.6. Characterization of Biogenically Synthesized AgNPs

The extracts were characterized before and after the addition of AgNO_3_. After confirming the formation of AgNPs, 3 mL of each extract was lyophilized to obtain a fine powder. The powder was then characterized using an FT-IR and ultraviolet-visible (UV-Vis) spectrophotometer. FT-IT spectra were acquired using the total reflection attenuation technique (TAR) with 16 scans and a resolution of 2 cm^−1^.

For AgNP production, the color change in the solution was evaluated along with a UV-Vis spectrophotometry reading with an absorption sweep from 200 to 700 nm, considering their respective characteristic SPR (around 400 nm to confirm the formation of AgNPs) [2,14,24,25,26].

The elemental composition of the AgNPs was determined by electron microscopy on a JSM-7610F microanalysis JEOL instrument operating at 2 Kv (JEOL Ltd., Tokyo, Japan), coupled with energy-dispersive X-ray spectroscopy (EDS). Morphology and particle size were analyzed by transmission electron microscopy (TEM, Hitachi 7700, Tokyo, Japan) operated at 100 kV. The samples were dispersed in water and applied to a lacey formvar-carbon supported copper grid (200 mesh size), and images were captured at 100, 50, and 20 nm scales.

### 2.7. Statistical Analysis

The relationships between the variables were initially explored via scatterplots, and pairwise correlations were evaluated. Gaussian process models were then constructed to predict outcomes $y\left(x\right)$ based on a p-dimensional vector of explanatory variables x∈X. The model takes the following form (Equation (1)):

(1) $${\left(y\left(x_{1}\right),\ldots ,y\left(x_{n}\right)\right)}^{T}|\mu ,\sigma^{2},R\sim MVN({\left(\mu \left(x_{1}\right),\ldots ,\mu \left(x_{n}\right)\right)}^{T},\sigma^{2}R),$$

where MVN denotes the multivariate normal distribution, $\mu \left(.\right)$ represents the mean function, $\sigma^{2}$ is an unknown variance parameter, and R is a correlation matrix. The scenarios considered were:

(1) Predicting “poly” (polyphenols), “proteins” (proteins), and “rate” (proteins/polyphenols) based on “day” (fungus age) and “hours” (hours of stress exposure).
(2) Predicting “wave” (wavelength), “area” (the area under the curve), and “absorb” (absorbance) based on “poly”, “proteins”, and “rate”.
(3) As in scenario (2), with additional explanatory variables “day” and “hours”.

Predictive performance was assessed in all cases by comparing the model predictions with the observed data. This was done by considering all available training data and using leave-one-out cross-validation. For numerical purposes, all variables were scaled between 0 and 1.

### 2.8. Assessing the Antibacterial Activity of AgNPs

The antimicrobial activity of the synthesized AgNPs was evaluated using the microdilution technique [27], with *E. coli* ATCC 8739 (Gram-negative) and *S. aureus* ATCC 6538 (Gram-positive) as model bacteria. Serial dilutions of the AgNPs were prepared (dilution factors of 2, 4, 8, 16, 32, 64, 128, 256, and 512) and added to a microplate. A bacterial inoculum, adjusted to a concentration of 0.5 on the McFarland scale (1.5 × 10^8^ bacterial cells mL^−1^), was added to each well from columns 1 to 11 of the microplate, with column 11 serving as the negative control (well with microbial growth) and column 12 as the positive control (well without microbial growth). The microplates were then incubated at 24 °C for 24 h.

As the McFarland scale relates turbidity to bacterial cell concentration, the antimicrobial activity was evaluated based on the difference in absorbance of each sample and its dilutions against the absorbance of the negative control after 24 h of incubation, assuming that an absorbance value of zero or lower than the value of the negative control represents the absence or low microbial cell growth. Finally, the Minimum Inhibitory Concentration (MIC) was defined as the highest dilution of AgNPs that inhibits bacterial growth. This was identified as the well with the lowest absorbance relative to the controls, following the M27-A3 protocol of the Clinical and Laboratory Standards Institute [28].

## 3. Results and Discussion

### 3.1. Growth Kinetics and Biomass Production of the Fungus R. stolonifer

Figure S1 in the Supporting Information shows the biomass production kinetics of the fungus *R. stolonifer* grown on PDA. The data shows non-linear behavior over time, with the maximum biomass recorded on day 3 at 0.180 g. Biomass ranged between 0.10 and 0.17 g, with no significant increase after day 3. This plateau in biomass production is attributed to the finite surface area and limited nutrients available on the Petri dish with PDA, leading to a maximum growth and biomass production point between days 2 and 3 [29].

The biomass obtained after day 3 corresponded with the appearance of sporangia, which is linked to the complete formation of their structure. Sporangia are the spore-bearing parts of the fungus, and their generation marks the transition to the exponential growth phase. The maximum biomass generation observed on day 3 was also related to rapid mycelial expansion and growth [30,31,32]. Consequently, it was determined that after day 3, spore production begins, signaling the start of a new growth phase.

Figure S1 also shows that the biomass production values exhibit larger error bars on some days. This behavior is primarily attributed to the individual responses of the fungus to incubation conditions, media, and growth phase [32,33]. The exponential phase of a filamentous fungus typically lasts from day 3 to day 5, entering the stationary phase during days 6 to 11. During the stationary phase, a fine black powder was observed on the surface, corresponding to the maturity or saturation of the sporangia. The sporangia usually break open, releasing spores with the black powder [29]. For the preparation of the extracts, the fungus was harvested on day 3, corresponding to the beginning of the exponential growth phase, when both structure and morphology are nearly complete. Additionally, day 11 was selected to mark the end of the stationary phase, when the sporangia are fully saturated with spores. These two stages enabled the evaluation of the effects of fungal maturity and spore influence on AgNP production and on the response of the extract to the stress conditions involved in the preparation.

### 3.2. Characterization of Fungus Extract

#### 3.2.1. Identification of Polyphenols in Fungus Extracts

Figure S2a in the Supporting Information shows the UV spectra of the fungus extracts after 3 days of growth. A signal around 260 nm increases in intensity from 2 to 48 h. Two additional signals are observed from 72 h onwards, increasing in intensity until 96 h. The single signal at approximately 260 nm is attributed to simple phenolic compounds, which typically present a single peak between 220 and 280 nm [34]. These compounds contain π-conjugated systems with hydroxy phenolic groups. After 72 h, the signal changes, suggesting that the phenolic groups are undergoing modifications, resulting in compounds derived from the simple phenolic structures.

Similar to the extracts after 3 days of growth (Figure S2a), Figure S2b shows that all spectra of the fungus extract after 11 days of growth exhibit a unique signal around 260 nm, which is attributed to simple phenolic compounds. According to Geană et al. [34], these compounds show a single peak between 220 and 280 nm due to π-conjugated systems with hydroxy phenolic groups. However, unlike the extracts from 3 days of growth, the signal in the 11-day extracts remains constant over time, only increasing in intensity. This suggests that the simple phenolic groups in the 11-day extracts are more stable [35].

**Figure 1:**
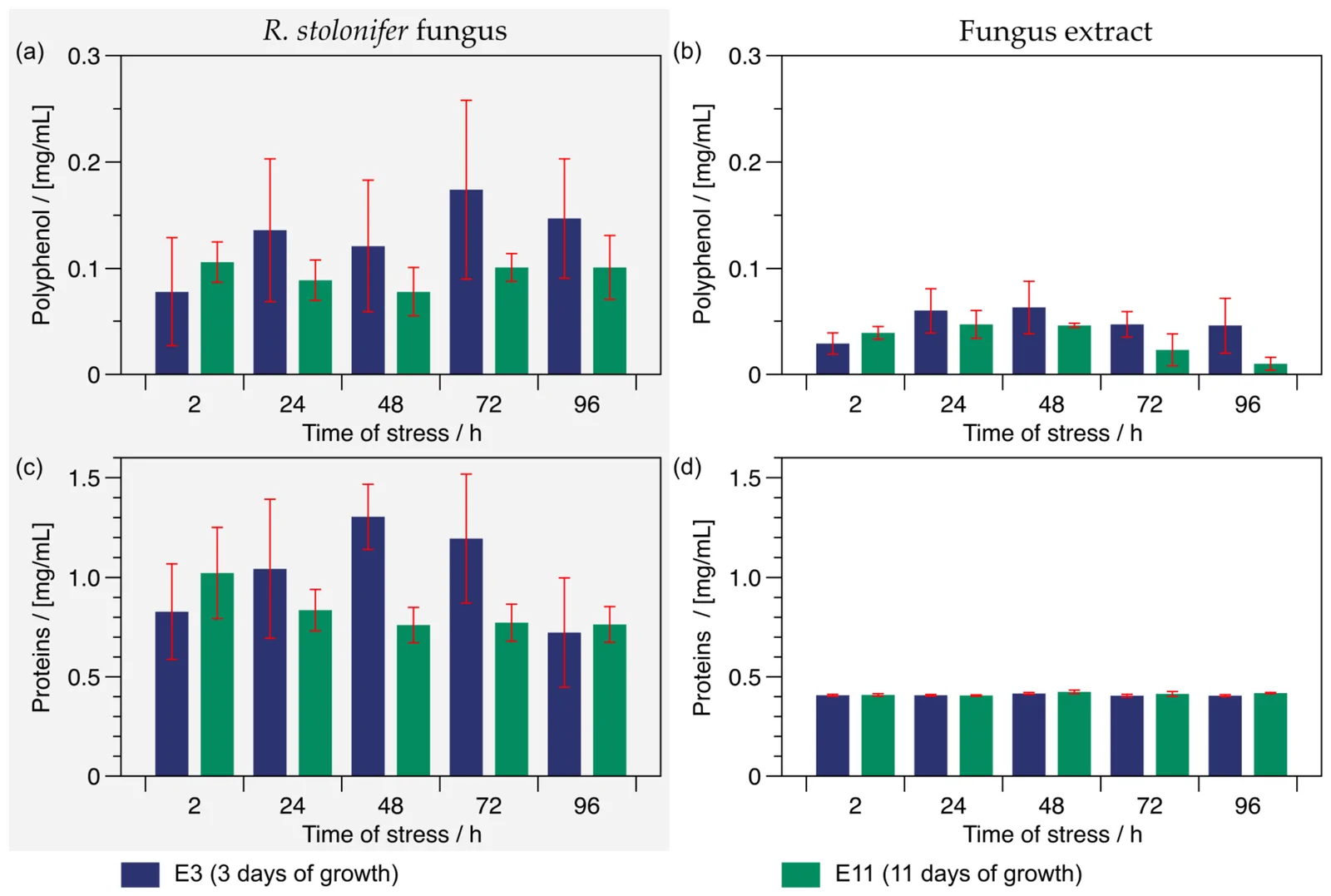
(**a**) Polyphenol concentration in extracts of the *R. stolonifer* fungus with 3 days-growth (E3) and 11 days-growth (E11); (**b**) polyphenol production in the fungus extract with 3 days of growth (E3) and 11 days of growth (E11) with respect to time; (**c**) protein concentration in the extracts of *R. stolonifer* fungus with 3 days-grown (E3) and 11 days-grown (E11); and (**d**) protein production in the fungus extract with 3 days of growth (E3) and 11 days of growth (E11) with respect to time. Note: E3: green; E11: blue.

Figure 1 show the results of quantifying protein and polyphenol concentrations in the fungus extracts from 3 days of growth (E3) and 11 days of growth (E11). For the reported polyphenol concentrations of different extracts (Figure 1a), the error bars are as large as the average values, sometimes even overlapping, indicating no significant differences between the concentrations obtained at different stress times. This variability between replicates is attributed to the individual responses of the fungus to the stress conditions. Polyphenols are generally produced in filamentous fungi as a response to environmental stress [35,36]. Furthermore, polyphenols provide adaptive advantages, helping fungi to adjust to specific environmental conditions and offering protection [37,38,39,40]. Based on the results obtained, it is hypothesized that the duration of stress does not directly determine the polyphenol concentration produced. The higher polyphenol concentration in extracts from E3 (3 days of growth) compared to E11 (11 days of growth) suggests that the fungus is more affected by stress in its early stages. In contrast, the lower polyphenol concentration in the E11 extract indicates that the fungus has adapted better to stress over time. Thus, the age of *R. stolonifer* plays a role in determining the polyphenol concentrations produced.

Figure 1b show the polyphenol concentrations produced, which were monitored at different time intervals (from 2 to 96 h), where it is evident that the production by the E3 fungus extracts is higher than that by E11 extracts. This trend can also be observed in the previous individual responses (Figure 1a), confirming that there is greater stress in young fungi. Furthermore, under both conditions, maximum production occurs between 24 and 48 h, suggesting that production decreases due to resource depletion and increased oxidative stress, which increases as time increases [38].

Statistical analysis of the reported results showed that, in the first case (Figure 1a), E3 shows a potential difference in average production (*p* = 0.054), while E11 exhibits no significant variation (*p* = 0.1454). In the second case (Figure 1b), E3 demonstrates a significant difference over time (*p* = 4.62e^−5^) compared to a less pronounced change in E11 (*p* = 0.093). Variance analysis of both conditions revealed more significant variability in E3 (*p* = 3.552e^−7^ and *p* = 0.00066), underscoring the dynamic stress response of younger fungi versus the stability of older fungi.

#### 3.2.2. Identification of Proteins in Fungus Extracts

Figure S3 presents the FT-IR spectra of the freeze-dried samples of *R. stolonifer* fungus extracts, where 9 distinct signals were identified across all spectra. Around 3280 cm^−1^ (line a in the figure), a broadband can be observed, which can be attributed to amine N-H bonds or peptide bonds. Lines b, c, and d, ranging from 2960 to 2850 cm^−1^, show several signals typically associated with asymmetric and symmetric C-H bond vibrations in methyl and methylene groups. The line e at approximately 1580 cm^−1^ corresponds to the symmetric deformation vibrations of the carboxylate groups (COO^−^). However, after 24, 48, and 72 h, this signal disappears, and two signals appear at 1636 and 1541 cm^−1^, corresponding to amide I and amide II, which are primarily due to the C–O and C–N stretching vibrations of the peptide’s main chain; this may be due to a rearrangement of the chains [41,42].

The f line, around 1400 cm^−1^, corresponds to the deformation vibrations of the C-H bonds in the methyl and methylene groups, as well as to the vibrations of the C=O bonds in the asymmetric carbonyl and carboxylate groups, which do not show significant differences in most of the extracts. The g line, located around 1250 cm^−1^, is attributed to the stretching of C-O bonds in phosphates or esters and exhibits greater intensity at 48 and 72 h. This may be due to the stress to which the fungus is subjected, causing it to begin producing a greater amount of these compounds during this time.

Line h, around 1070 cm^−1^, is typically linked to both C-O and C-C bond stretching and C-N bond vibrations [43,44] (which can be confirmed due to the sp3 hybridization of these bonds). Finally, line i, located around 650 cm^−1^, is related to out-of-plane vibrations of N-H and C=O bonds. The collective presence of these signals, particularly those indicated by lines a, b, c, e, g, h, and i, suggests the presence of functional groups common in proteins, fatty acids, and lipids [45]. Therefore, it can be inferred that the extracts contain substantial concentrations of these biomolecules.

The fungus *R. stolonifer*, like many fungi, produces stress-response proteins that aid cells in surviving under adverse conditions. For example, these proteins neutralize free radicals and prevent oxidative damage [46]. Heat shock proteins (HSPs) are also generated to protect other proteins from denaturation and assist in proper protein folding under heat stress conditions [47]. Moreover, proteins involved in producing secondary metabolites may have defensive or signaling functions [37]. These findings suggest that protein production varies significantly depending on the duration or exposure to stress.

Figure 1c illustrates the protein concentration in the different extracts from E3 and E11 (2, 24, 72, and 96 h), showing no significant differences between these time points, as was the case in the results for polyphenols. This suggests that stress exposure duration may influence protein and polyphenol production in *R. stolonifer*, as the stress response is highly variable and depends on both the specific stress condition and the fungal maturity stage [35]. For example, protein production in E11 is generally lower than in E3 at most stress time points, indicating that E3 exhibits a higher protein production response to stress with greater variability than E11. This difference is attributed to the distinct growth and maturity stages of the fungus. More mature stages, such as E11, tend to respond more consistently to stress, while less mature stages, such as E3, show greater variability in their metabolic responses, reflecting higher vulnerability to stress [29,30].

When monitoring protein production over time for extracts E3 and E11 (Figure 1d), both conditions exhibit similar trends over time, with a peak in production between 48 and 72 h. Regarding polyphenol and protein production under stress conditions caused by nutrient scarcity and constant agitation, filamentous fungi adjust their metabolic processes to optimize survival [35]. Proteins, essential for critical cellular functions such as repair and maintenance of cell structure, tend to remain stable as the fungus prioritizes their production to cope with stress [36]. In contrast, polyphenols, which serve as antioxidants and provide protection against oxidative damage, may be reduced. Under these stress conditions, polyphenol synthesis is diverted as a secondary metabolic pathway, redirecting resources towards protein production and maintenance [30,39]. This adjustment enables the fungus to concentrate its limited resources on the essential components required for short-term adaptation and survival.

Regarding our statistical analysis of protein quantification for the first case (Figure 1c), E3 exhibits significant differences in average production over time (*p* = 0.0025), indicating dynamic fluctuations, while E11 shows less pronounced changes (*p* = 0.019). In the second condition (Figure 1d), neither E3 (*p* = 0.10) nor E11 (*p* = 0.3382) shows significant temporal differences in average protein production. However, variance analysis revealed consistently higher variability in E3 compared to E11, with significant *p*-values (4.24 × 10^−5^ and 2.25 × 10^−9^, respectively). These findings underscore the dynamic and variable stress response in younger fungi, in contrast to the stable and consistent protein production in older fungi across different conditions.

#### 3.2.3. Characterization of Compounds in the Fungus Extracts

The chemical profile of the *R. stolonifer* extract (E3) was characterized using GC-MS to identify the specific metabolites involved in the biogenic synthesis of AgNPs (Figure S4). The chromatogram revealed a complex, multicomponent system consisting of approximately 100 different metabolites (match score > 80, Figures S5–S7). This mixture of distinct chemical families, summarized in Table 1, includes phenolic compounds, fatty acids and organic esters, terpenoids, and heterocyclic species. These results provide a characterization of the fungal secretome and offer essential insights into the dual-action mechanism of reduction and stabilization.

Unlike plant-mediated synthesis—where high concentrations of complex flavonoids or xanthones often act as both reducing and capping agents [48,49,50], fungal extracellular synthesis suggests a distinct synergistic mechanism [15,28,51]. The analysis undertaken identified a series of simple phenolic species such as phenol (8.9 min), p-cresol (11.7 min), guaiacol (11.9 min), and 4-vinylguaiaicol (18.4 min), alongside polysubstituted phenols such as catechol (1,2-benzenediol, 15.4 min), hydroquinone (1,4-dihydroxybenzene, 17.6 min), 4-hydroxybenzyl alcohol (19.5 min), 2,3-dihydroxybenzoate (26.4 min), resorcinol (1,3-dihydroxybenzenes, 10.5 min), 2,3-dihydroxybenzoate (24.6 min). These molecules feature π-conjugated systems with multiple hydroxyl groups, which correlate with the UV-Vis absorbance at 265 nm and serve as the primary reducing agents facilitating the electron transfer from Ag^+^ to Ag^0^ [50,52,53].

To validate the specific role of these compounds, standard gallic acid and ferulic acid as formers of silver NPs were tested. As reported elsewhere, these compounds can reduce silver ions but fail to produce stable NPs under controlled conditions [50,54]. This suggests that while simple phenols possess the redox potential to reduce silver, they lack the steric bulk and specific functional groups necessary to ensure long-term colloidal stability on their own. Therefore, the successful formation of AgNPs in an *R. stolonifer* system is attributed to the immediate reduction of silver ions by the phenolic mixture on contact. Once the silver atoms begin to nucleate into nanoparticles, the stabilization process is mediated by methyl and/or prenyl groups, primarily provided by the proteins and methoxylated phenols present in the fungal extract [15,40,50,54]. This highlights a critical functional synergy where phenolic hydroxyls drive the initial reduction, while the methyl-rich protein fraction provides the necessary capping properties to prevent metal aggregation [51,55,56]. This relationship between polyphenol concentration and protein structure is fundamental to understanding how *R. stolonifer* can achieve efficient biogenic synthesis [39,46].

### 3.3. Biogenic Synthesis of AgNPs

Table 2 presents data for selected E3 and E11 fungus extract samples, highlighting that the SPR occurs within the wavelength range of 430 to 490 nm. This variation in SPR is related to the interaction with organic matter. As previously reported, although the SPR for AgNPs typically occurs at 400 nm in biogenic synthesis, the UV-Vis signal falls within the range of 430–470 nm [55]. The SPR analysis partially confirms the formation of AgNPs but also reveals a lack of uniformity across the replicates. It was evident that the formation of nanoparticles was not influenced by the reaction time but by the characteristics of each extract, which in turn affected nanoparticle formation. Based on these findings, it was determined that there is a relationship between the extract characteristics under stress (i.e., polyphenols, proteins and amino acid concentrations) and their effect on the formation and properties of AgNPs [57,58,59].

After all quantification data and results from the synthesis of AgNPs were compiled and optimal ratio ranges were considered, the samples were ranked in descending order based on their protein/polyphenol ratio (Table 2). Table 2 indicates that, in some cases, despite the ratio being adequate, the results of nanoparticle production are negative.

A further analysis was conducted to determine optimal conditions. Three key conditions were stipulated for AgNP formation with *R. stolonifer* fungus extracts: (a) polyphenols concentration should range from 0.120 to 0.024 mg mL^−1^; (b) protein concentration should range from 0.947 to 0.399 mg mL^−1^; and (c) the protein/polyphenol ratio should range from 5 to 33. Table 1 highlights, in bold, all data that do not meet these three conditions, mostly representing samples that did not lead to the formation of AgNPs.

#### 3.3.1. Characterization of the Biogenic AgNPs

The AgNPs produced using fungus extracts were classified according to the plasmon position in each sample. The use of UV-Vis spectroscopy is a fundamental technique for observing nanoparticle formation through SPR at a specific wavelength [24,60,61]. Additionally, the wavelength, absorbance, and area under the SPR curve provide insights into the size and shape of the AgNPs, as their size influences the intensity and position of the SPR due to the volume-to-surface area ratio. Similarly, the shape of the AgNPs affects how electrons respond to the incident electromagnetic field, resulting in different resonance modes depending on the geometry [49].

As shown in Figure S8, the signals share the same range with different absorbances and overlap, indicating that the NPs must be morphologically similar. For their characterization, several samples were selected from each range: range 1 (Figure S8), which spans from 435 to 425 nm, sample S3-524 was chosen; range 2 (Figure S9), covering the interval from 455 to 446 nm, samples S3-24a, S11-24c, S11-48c, and S3-72a were selected; range 3 (Figure S10), covering 445 to 437 nm, samples S3-320, S11-48d, E11-96d, and S11-827 were chosen; and, range 4 (Figure S11), which spans from 435 to 425 nm, samples S3-430 and S11-824 were selected. The selected samples were characterized using HRTEM and EDS to correlate their morphology and size with the reported plasmon shape.

Figure S12 presents the FI-TR spectrum for the fungus extract samples and the synthesized AgNPs. Notable signals denoted as a, b, and c were observed, where Signal a at 1580 cm^−1^ is typically associated with the stretching vibrations of the C=C (sp2 hybridization) bond in aromatic rings or with the vibrations of the amide group (NH_2_-C=O) [56]. Since fungus extracts contain proteins and aromatic compounds, this band suggests that these components may play a role in reducing and stabilizing AgNPs. Signal b at 1472 cm^−1^ was attributed to the angular deformation of CH_2_ or CH_3_ bonds found in lipids or the vibrations of methyl and methylene groups in polysaccharides. These functional groups are common in fungal extracts, and their presence in the FT-IR spectrum suggests that lipids and polysaccharides are involved in the formation and stabilization of nanoparticles [26]. Finally, signal c at 1072 cm^−1^ can be attributed to C-O stretching vibrations in alcohols, ethers, or possibly to C-N stretching vibrations in amines. Secondary metabolites in fungal extracts, such as carbohydrates and proteins, typically exhibit these bonds. The presence and enhancement of this signal suggest that these organic components of the fungal extract actively contribute to the synthesis of AgNPs [52]. The increase in the FT-IR spectrum peaks further confirms the involvement of organic compounds in the synthesis process.

Table S1 presents the elemental analysis of the synthesized AgNPs, revealing significant variability in the percentages of silver (%Ag) and oxygen (%O) among the different samples. The %Ag values ranged from 7% to 95%, indicating a wide concentration range likely influenced by the synthesis conditions and experimental parameters. On the other hand, an inverse relationship can be observed between the percentages of silver and oxygen. Specifically, samples with higher silver content tend to have lower oxygen concentrations.

The above-noted inverse relationship suggests that a high concentration of silver in the nanoparticles could be related to reduced oxide formation due to competition between silver and oxygen during synthesis [62]. As the amount of available silver increases, its reduction and stabilization in the form of nanoparticles is likely to be favored, which limits the interaction of oxygen with biomolecules and consequently reduces the formation of metal oxides. This inverse relationship between %Ag and %O provides an important insight for tuning nanoparticle composition and physicochemical properties. Understanding this balance is fundamental to designing nanoparticles with specific characteristics, such as increased stability, controlled reactivity, or enhanced properties for antimicrobial applications, and ultimately allowing their optimization. All samples resulted in spherical-shaped NPs with a wide distribution in size. Additionally, a biofilm of organic matter surrounding the particles was noted, which aligns with the observation from previous studies.

As shown in Table 3 and Figure S13, Range 1 exhibits a high variability in particle size and a high polyphenols/proteins ratio, particularly for the larger sizes. In this range, large-sized NPs correlate with high polyphenol/protein ratios, suggesting that NPs size may influence the relative concentration of these components. In contrast, Ranges 2 and 3 show relatively small and medium NP sizes, with significantly lower polyphenols/protein ratios compared to Range 1. Specifically, in Range 2, smaller NP sizes are associated with lower ratios, while in Range 3, intermediate sizes do not show a clear pattern in the polyphenols/protein ratio. These results suggest that NP size can significantly impact the polyphenols/protein ratio, particularly for the larger sizes. It can be concluded that a high protein/polyphenol ratio favors the growth of large particles. Therefore, range classification does not significantly correlate with size, indicating that other factors also influence the synthesis kinetics. These findings highlight the importance of considering multiple variables in optimizing the NP formation process and suggest that specific characterizations are crucial to evaluate their size, morphology, and dispersion as a function of the synthesis conditions.

#### 3.3.2. Process Optimization

**Figure 2:**
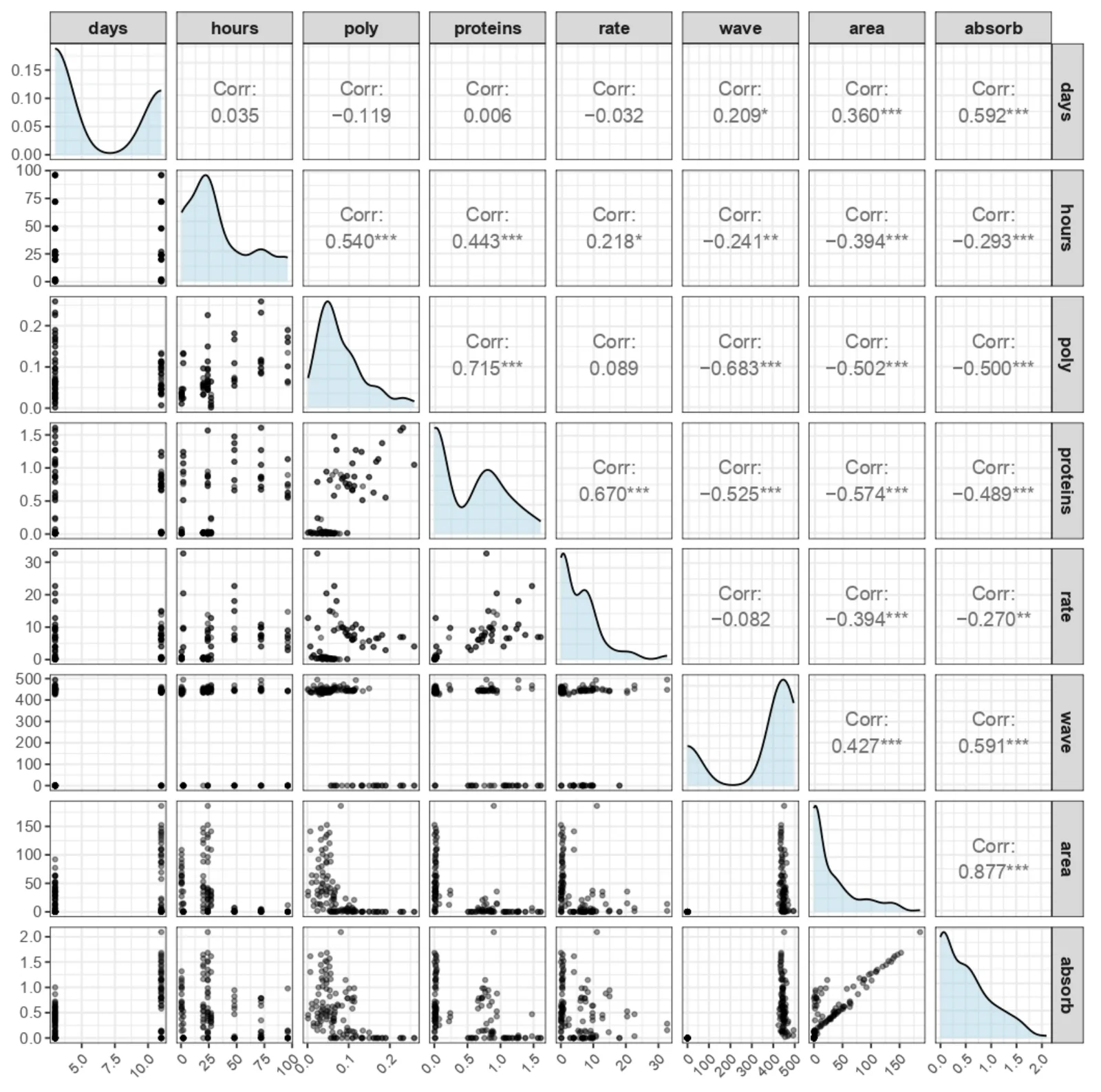
Correlations and scatterplots for pairwise relationships between variables. *** Highly significant correlation i.e., *p* < 0.001, ** Very significant correlation i.e., *p* < 0.01, * Statistically significant correlation i.e., *p* < 0.05. Marginally significant correlation *p* < 0.10, No symbol means correlation is not significant i.e., *p* >= 0.10.

As evident from the experimental results, the processes involve significant variability in microorganisms present, making process optimization complex. A feasible approach is to investigate correlations between the variables, as can be observed in Figure 2, which provides a scatter plot of all the variables. Explanatory variables can be useful for prediction and for optimization. For example, a linear relationship between hours of stress and polyphenol concentration can be seen, while a strong relationship between wavelength and any other variable is not evident. Although correlations exist, they should be interpreted cautiously, as they are only significant if the pairwise relationship is approximately linear.

**Figure 3:**
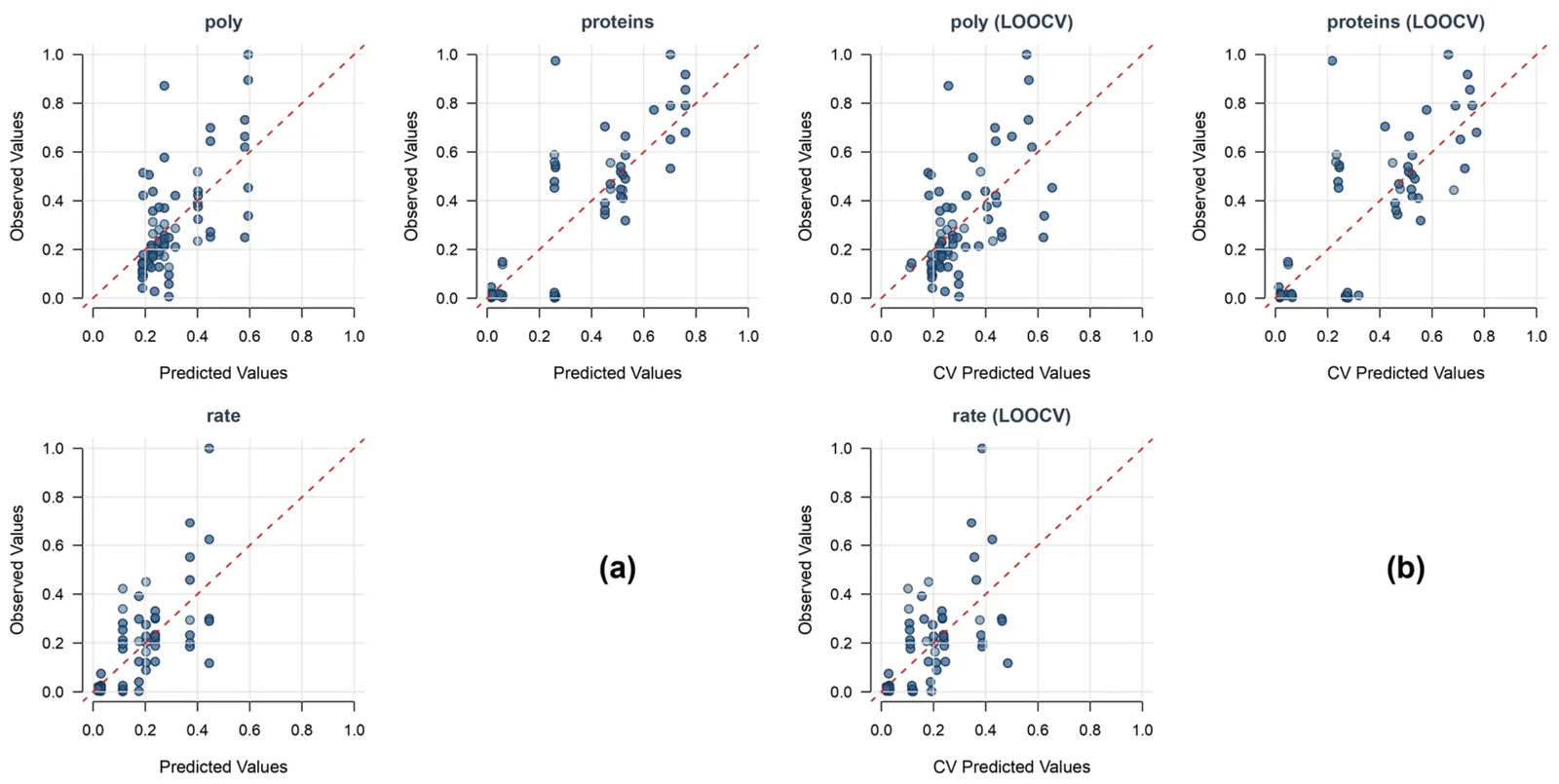
Relationships between observed data and model predictions for polyphenols (poly), proteins (proteins) and protein/polyphenol rate (rate): (**a**) for all data considered for training and (**b**) when leave-one-out cross-validation was used.

**Figure 4:**
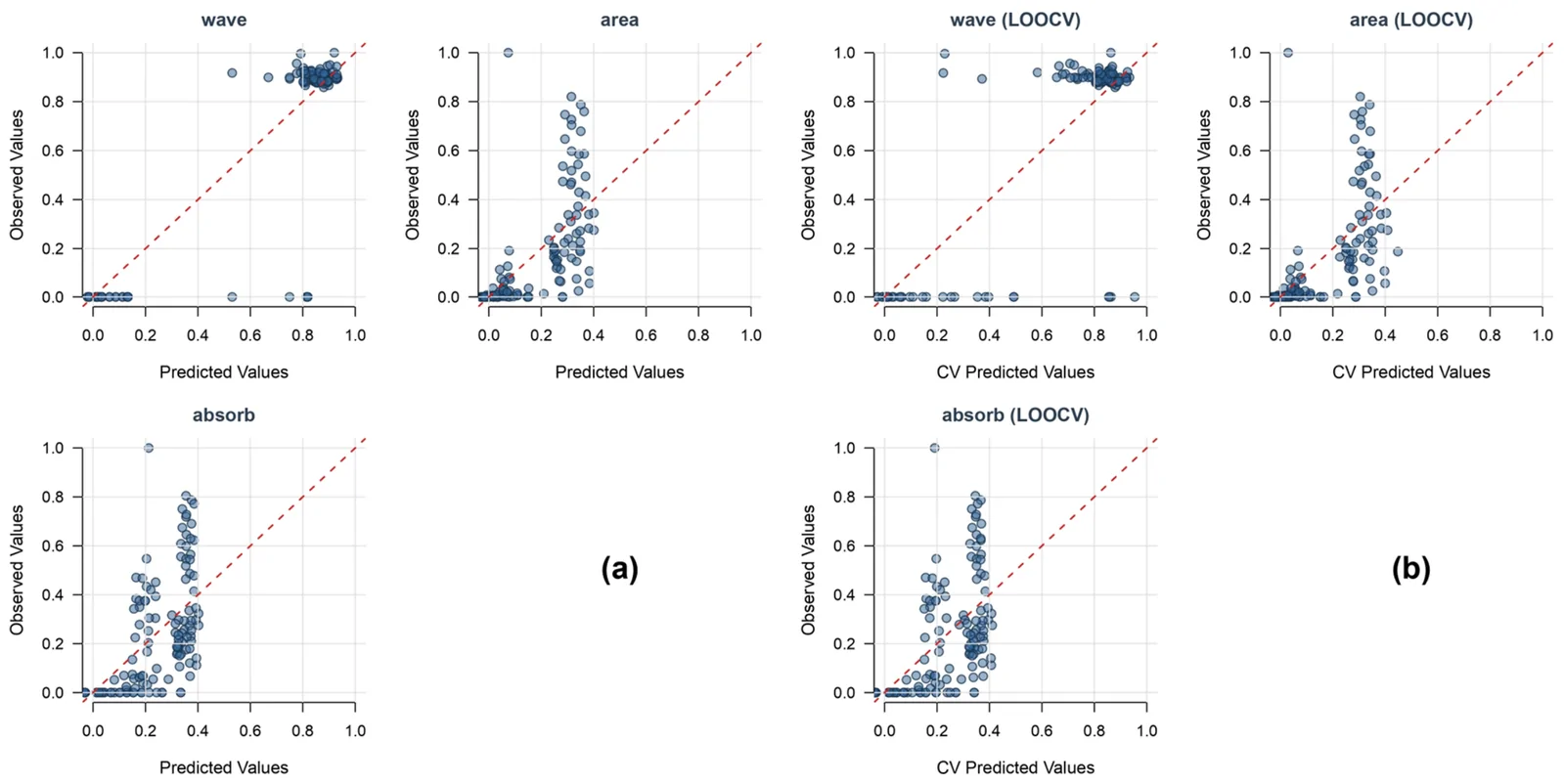
Relationships between the observed data and the model predictions for wavelength (wave), area under the curve (area) and absorbance (absorb): (**a**) all data were considered for training and (**b**) when leave-one-out cross-validation was used.

**Figure 5:**
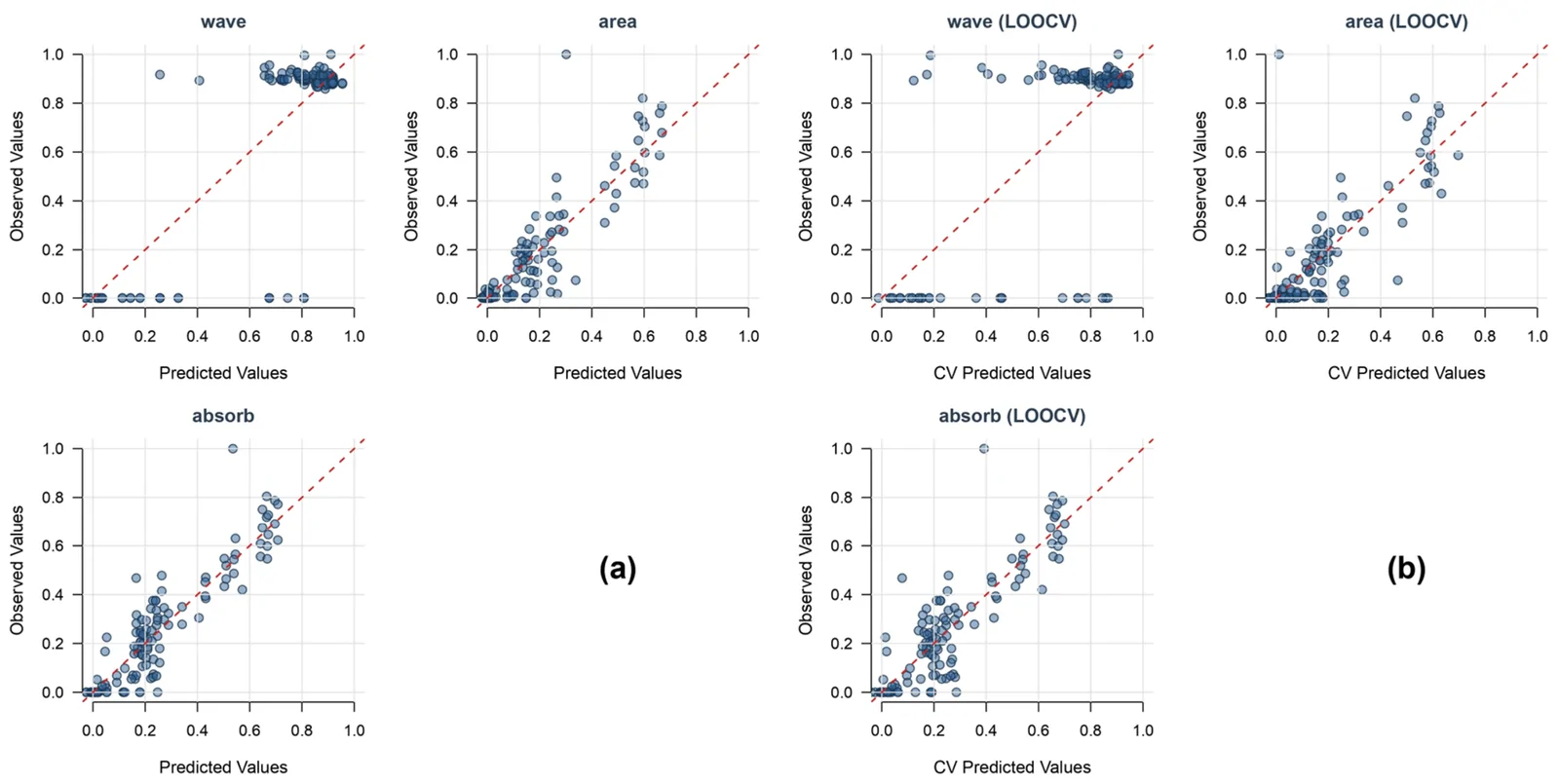
Relationships between the observed data and the model predictions for wavelength (wave), area under the curve (area) and absorbance (absorb): (**a**) when all data were considered for training and (**b**) when leave-one-out cross-validation was used.

Figure 3, Figure 4 and Figure 5 display the predictive performance of the developed Gaussian process models for the three scenarios described in Section 2.7. The first plot given in each figure represents predictions using all data for training, while the second plot shows predictions based on leave-one-out cross-validation. Notably, predictive performance in the latter case is generally expected to be lower, as predictions made from training data are typically more accurate than those used for validation.

In Figure 3, a potentially useful predictive model is presented for proteins, as there is strong agreement between the observed data and the model predictions (R^2^ = 0.81). For polyphenol and protein/polyphenol ratio, some agreement can be observed between the data and predictions, though this relationship is more variable than that for proteins (R^2^ = 0.57 and 0.65, respectively). Figure 4 shows generally poor predictive performance across outcomes. For wavelength, the model can predict whether the observed data are relatively large or not (resulting in a correlation of 0.78) but has limited predictive ability otherwise. A weak relationship exists between observed and predicted values (R^2^ = 0.63 and 0.54, respectively) for the area under the curve and absorbance of the UV-Vis spectra. When additional explanatory variables are considered (Figure 5), the predictive models for the area under the curve and absorbance of the UV-Vis spectra improve substantially, with stronger relationships between observed and predicted values (R^2^ = 0.81 and 0.87, respectively). This suggests that incorporating additional data, such as fungus age and stress hours, improves the predictability of the outcomes.

### 3.4. Antibacterial Efficacy of AgNPs

All disinfection tests showed a higher disinfection percentage than the microbial culture without any disinfectant (i.e., negative control). As shown in Figure S14, most AgNPs show 100% disinfection, which means the elimination of microbial growth after 24 h, indicating high disinfection activity up to a dilution factor of 4 for *E. coli* and up to a dilution factor of 8 for *S. aureus*. Above a dilution factor of 8, the disinfection values against *E. coli* increase sharply, which is typical due to the higher structural resistance of *E. coli* compared to *S. aureus* [63], meaning that the AgNPs have a minimum inhibitory concentration (MIC) at dilution factor 4 for Gram-negative model bacteria in all samples and a MIC at dilution factor 8 for Gram-positive model bacteria for all samples except sample S3-524.

This may be because Gram-positive bacteria have a thick layer of peptidoglycan and lack an outer membrane, while Gram-negative bacteria have a thin layer of peptidoglycan and possess a complex outer membrane containing lipopolysaccharide [64]. This makes Gram-positive bacteria more susceptible to inactivation than Gram-negative bacteria. On the other hand, this can also be observed through the mechanism of inactivation of Gram-positive bacteria, since during the diffusion phase, the nanoparticles concentrate largely in the extracellular space, infiltrating the cell wall as the concentration inside increases, causing them to distribute throughout the cell wall and the cytoplasmic membrane, resulting in a higher intracellular concentration [63]. In the diffusion phase of the mechanism for inactivating Gram-negative bacteria, the nanoparticles are highly concentrated in the extracellular space, infiltrating the outer membrane and exhibiting a decreasing concentration gradient from the outside towards the inside. They are distributed throughout the periplasmic space, indicating an equilibrium concentration between the extracellular and intracellular environments [63]. However, even at a dilution factor of 512, most NPs maintained a disinfection value above that of the negative control, indicating that bacterial growth was still prevented. In contrast, exceptions were observed for *S. aureus*, where some samples in range 2 and 3, namely S11-824, S3-320, S11-827, and S11-830, showed higher bacterial growth at dilutions of 128 and 512. This suggests that at low concentrations, certain AgNP formulations may act as a nutrient for *S. aureus* or may not generate sufficient oxidative stress to inhibit their growth, allowing the bacteria to adapt [64]. This phenomenon requires further investigation.

## 4. Conclusions

Based on the results obtained from the synthesis of silver nanoparticles with *R. stolonifer* fungus extracts, the following conclusions were reached:

Fungi act in a way that their stress response and biomolecule production are not directly dependent on time or specific stress conditions.

The differences in the structure and maturity of the fungi between day 3 and day 11 are significant. The fungus has greater stability on day 11 and better adaptation to stress conditions, allowing for the production of biomolecules with low variability between replicates.

No significant differences were observed between fungi during the synthesis of silver nanoparticles (AgNPs), suggesting that synthesis conditions can be standardized to obtain consistent results based on biomolecule production.

The optimal conditions for the synthesis of AgNPs with *Rhizopus stolonifer* fungus extracts require adequate concentrations of proteins and polyphenols and strong interactions between these components, with efficient synthesis when the proportion of proteins is 5 to 33 times higher than that of polyphenols.

All nanoparticles proved effective in the disinfection of *E. coli* and *S. aureus* regardless of their UV-vis range (different morphology and size), achieving 100% disinfection with dilution factors up to 4 and 8, respectively, irrespective of the differences between AgNPs.

Although the response of fungi to stress may vary depending on their maturity, the optimal conditions for the synthesis of AgNPs are the ratio and concentration of proteins and polyphenols, with the resulting nanoparticles being highly efficient in bacterial disinfection despite morphological differences present, underlining the feasibility of this methodology to produce effective antimicrobial agents.

## Figures and Tables

**Table 1:** Characterization of *R. stolonifer* extracts with a total of 130 compounds detected with a criterion of match score ≥ 80.

No.	Retention Time	Compound Name	CAS#	Formula	Molecular Weight (Da)	Match Factor	Area
1	4.95	furfural	9998-01-1	C_5_H_4_O_2_	96.08	98.6	5.7 × 10^5^
2	7.21	1,2-cyclopentanedione	003008-40-0	C_5_H_6_O_2_	98.1	97.9	9.9 × 10^5^
3	8.28	5-methylfurfural	99 620-02-0	C_6_H_6_O_2_	110.11	97.9	7.8 × 10^5^
4	1.91	acetic acid	000064-19-7	C_2_H_4_O_2_	60.05	97.6	5.2 × 10^6^
5	6.85	gamma-butyrolactone	7331-52-4	C_4_H_6_O_2_	86.09	97.6	3.0 × 10^6^
6	1.78	acetaldehyde, hydroxy-	000141-46-8	C_2_H_4_O_2_	60.05	97.3	9.1 × 10^6^
7	1.58	formic acid	000064-18-6	CH_2_O_2_	46.03	97.2	2.0 × 10^6^
8	4.78	2-methylpyrazine	109-08-0	C_5_H_6_N_2_	94.11	96.9	2.2 × 10^5^
9	16.09	5-hydroxymethylfurfural	000067-47-0	C_6_H_6_O_3_	126.11	96.9	9.3 × 10^6^
10	2.36	1-hydroxy-2-propanone	116-09-6	C_3_H_6_O_2_	74.08	96.8	1.9 × 10^6^
11	8.93	phenol	108-95-2	C_6_H_6_O	94.11	96.2	2.1 × 10^6^
12	18.36	4-vinylguaiacol	7786-61-0	C_9_H_10_O_2_	150.17	94.9	5.5 × 10^5^
13	1.71	isobutyraldehyde	78-84-2	C_4_H_8_O	72.11	94.8	1.7 × 10^5^
14	8.83	2,4-dihydroxy-2,5-dimethyl-3(2H)-furan-3-one	010230-62-3	C_6_H_8_O_4_	144.13	94.7	1.3 × 10^6^
15	19.46	p-hydroxybenzyl alcohol	623-05-2	C_7_H_8_O_2_	124.14	94.5	1.4 × 10^6^
16	3.73	1-hydroxy-2-propanone	116-09-6	C_3_H_6_O_2_	74.08	93.6	2.5 × 10^6^
17	25.41	uracil	66-22-8	C_4_H_4_N_2_O_2_	112.09	93.5	3.6 × 10^6^
18	11.94	o-methoxyphenol	90-05-1	C_7_H_8_O_2_	124.14	93.3	2.7 × 10^5^
19	2.29	isovaleraldehyde	590-86-3	C_5_H_10_O	86.13	92.9	2.3 × 10^5^
20	2.08	oxolane	000000-00-0	C_4_H_8_O	72.11	92.7	4.2 × 10^4^
21	11.70	para-cresol	000000-00-0	C_7_H_8_O	108.14	92.2	8.3 × 10^5^
22	9.16	2H-pyran-2,6(3H)-dione	005926-95-4	C_5_H_4_O_3_	112.08	92.1	3.7 × 10^5^
23	13.66	4H-pyran-4-one, 2,3-dihydro-3,5-dihydroxy-6-methyl-	028564-83-2	C_6_H_8_O_4_	144.12	92.1	3.5 × 10^6^
24	15.44	1,2-benzenediol	120-80-9	C_6_H_6_O_2_	110.11	92	1.5 × 10^6^
25	2.68	2,3-pentanedione	000000-00-0	C_5_H_8_O_2_	100.12	91.3	1.1 × 10^5^
26	11.10	4-hydroxy-2,5-dimethyl-3(2h)-furanone	000000-00-0	C_6_H_8_O_3_	128.13	91.3	3.9 × 10^5^
27	3.01	methacrylic anhydride	000760-93-0	C_8_H_10_O_3_	154.16	91.1	4.3 × 10^4^
28	23.35	beta-d-glucopyranose, 1,6-anhydro-	000498-07-7	C_6_H_10_O_5_	162.14	91	4.0 × 10^6^
29	10.33	2,5-furandione, 3,4-dimethyl-	000766-39-2	C_6_H_6_O_3_	126.11	90.6	8.9 × 10^4^
30	15.88	dihydrobenzofuran	496-16-2	C_8_H_8_O	120.15	90.6	1.7 × 10^6^
31	28.84	4-hydroxyphenylacetamide	017194-82-0	C_8_H_9_NO_2_	151.16	90.5	2.9 × 10^6^
32	2.37	butanal, 2-methyl-	000096-17-3	C_5_H_10_O	86.13	90.4	2.5 × 10^5^
33	7.74	2,5-furandione, 3-methyl-	000616-02-4	C_5_H_4_O_3_	112.08	90.4	2.7 × 10^5^
34	14.13	2-cyclopenten-1-one	000930-30-3	C_5_H_6_O	82.10	90.1	1.0 × 10^5^
35	3.42	2,2′-bioxirane	001464-53-5	C_4_H_6_O_2_	86.09	88.4	1.5 × 10^6^
36	4.72	2(5h)-furanone, 3-methyl-	022122-36-7	C_5_H_6_O_2_	98.1	88.3	1.4 × 10^5^
37	57.10	dilauryl thiodipropionate	123-28-4	C_30_H_58_O_4_S	514.84	87.9	8.7 × 10^5^
38	3.18	pyrazine	000290-37-9	C_4_H_4_N_2_	80.09	87.5	1.9 × 10^4^
39	12.54	furan	110-00-9	C_4_H_4_O	68.07	87.4	2.6 × 10^5^
40	4.06	propanoic acid, 2-oxo-, methyl ester	000600-22-6	C_4_H_6_O_3_	102.09	87.3	1.0 × 10^6^
41	5.45	2-furanmethanol	000098-00-0	C_5_H_6_O_2_	98.1	87.3	2.3 × 10^6^
42	10.52	5-methylfurfural	620-02-0	C_6_H_6_O_2_	110.11	87.1	1.3 × 10^5^
43	17.62	1,4-dihydroxybenzene	123-31-9	C_6_H_6_O_2_	110.11	87	2.0 × 10^6^
44	20.90	1, 1, 5-trimethyl-1, 2-dihydronaphthalene	30364-38-6	C_13_H_16_	172.27	86.9	9.4 × 10^4^
45	20.52	toluene	108-88-3	C_7_H_8_	92.14	86.7	4.4 × 10^4^
46	6.11	4-cyclopentene-1,3-dione	000930-60-9	C_5_H_4_O_2_	96.08	86.6	6.1 × 10^5^
47	10.63	1,3-dioxol-2-one,4,5-dimethyl-	037830-90-3	C_5_H_6_O_3_	114.1	86.6	9.2 × 10^5^
48	5.78	acetol acetate	000000-00-0	C_5_H_8_O_3_	116.12	86.4	8.4 × 10^4^
49	19.86	propanoic acid, 3-chloro-, 4-formylphenyl ester	000000-00-0	C_10_H_9_ClO_3_	212.63	86.3	1.7 × 10^5^
50	22.65	4-tolyl isocyanate	622-58-2	C_8_H_7_NO	133.05	86.3	5.2 × 10^5^
51	11.55	2-furancarboxylic acid	000088-14-2	C_5_H_4_O_3_	112.08	86	3.6 × 10^5^
52	16.65	acetic anhydride	000108-24-7	C_4_H_6_O_3_	102.09	85.9	2.8 × 10^6^
53	10.72	ethylene glycol diacetate	000000-00-0	C_6_H_10_O_4_	146.14	85.6	6.5 × 10^5^
54	29.63	adenine	000073-24-5	C_5_H_5_N_5_	135.13	85.6	1.3 × 10^6^
55	2.69	propenoic acid	79-10-7	C_3_H_4_O_2_	72.06	85.4	3.0 × 10^5^
56	6.70	2,2′-bifuran, 2,2′,5,5′-tetrahydro-	098869-92-2	C_8_H_10_O_2_	138.16	84.9	2.1 × 10^5^
57	20.77	4-pyridinecarboxamide	001453-82-3	C_6_H_6_N_2_O	122.13	84.6	1.9 × 10^5^
58	2.60	propionic acid	000000-00-0	C_3_H_6_O_2_	74.08	84.5	6.8 × 10^4^
59	33.74	norharmane, n-acetyl-	1011-37-6	C_13_H_10_N_2_O	210.23	84.5	3.2 × 10^5^
60	5.76	proline, 2-methyl-5-oxo-, methyl ester	056145-24-5	C_7_H_11_NO_3_	157.17	84.4	4.8 × 10^4^
61	23.69	butyl para-hydroxybenzoate	94-26-8	C_11_H_14_O_3_	194.23	84.3	3.0 × 10^5^
62	14.24	heptanediamide, n,n′-di-benzoyloxy-	10250-13-2	C_21_H_22_N_2_O_6_	398.41	84.1	9.6 × 10^4^
63	3.66	3-methylpyridazine	1632-76-4	C_5_H_6_N_2_	94.11	84	1.1 × 10^5^
64	14.72	4h-pyran-4-one, 3,5-dihydroxy-2-methyl-	1073-96-7	C_6_H_6_O_4_	142.11	84	4.8 × 10^5^
65	11.90	4-hydroxy-2,5-dimethyl-3(2h)-furanone	3658-77-3	C_6_H_8_O_3_	128.13	83.3	9.9 × 10^5^
66	8.05	2-furanmethanol, 5-methyl-	3857-25-8	C_6_H_8_O_2_	112.13	83	2.4 × 10^5^
67	19.47	benzenemethanol, 3-hydroxy-	620-24-6	C_7_H_8_O_2_	124.14	82.5	2.4 × 10^6^
68	16.64	phenylacetic acid	103-82-2	C_8_H_8_O_2_	136.15	82.3	2.6 × 10^5^
69	22.23	naphthalene, 1,2-dihydro-4,5,7-trimethyl-	53156-11-9	C_13_H_16_	172.27	82.3	4.7 × 10^4^
70	11.56	3h-1,2,4-triazol-3-one, 1,2-dihydro-	930-33-6	C_2_H_3_N_3_O	85.06	82.2	2.4 × 10^5^
71	29.92	1-undecene, 9-methyl-	74630-41-4	C_12_H_24_	168.32	82.1	8.6 × 10^4^
72	1.30	(2-aziridinylethyl)amine	4025-37-0	C_4_H_10_N_2_	86.14	82	9.3 × 10^6^
73	17.07	2-fluoroimidazole	57212-34-7	C_3_H_3_FN_2_	86.07	82	7.0 × 10^4^
74	14.13	2-cyclopenten-1-one	930-30-3	C_5_H_6_O	82.1	81.7	7.6 × 10^4^
75	7.74	furan	110-00-9	C_4_H_4_O	68.07	81.5	2.5 × 10^5^
76	19.88	bis(2-ethoxyethyl) ether	112-36-7	C_8_H_18_O_3_	162.23	81.4	8.0 × 10^4^
77	16.65	ketone, methyl 2-methyl-1,3-oxothiolan-2-yl	5684-35-1	C_6_H_10_O_2_S	146.21	81.3	4.3 × 10^6^
78	19.86	2-(2-methoxyethoxy)ethanol	111-77-3	C_5_H_12_O_3_	120.15	81.2	1.3 × 10^5^
79	9.42	1h-pyrrole-2,5-dione	541-59-3	C_4_H_3_NO_2_	97.07	81.1	7.4 × 10^4^
80	25.18	pyrolo[3,2-d]pyrimidin-2,4(1h,3h)-dione	496-04-8	C_6_H_5_N_3_O_2_	151.12	80.7	1.1 × 10^5^
81	1.53	cyclobutylamine	2516-34-9	C_4_H_9_N	71.12	80.1	3.6 × 10^6^
82	12.87	.alpha.-l-galactopyranoside, methyl 6-deoxy-	14687-15-1	C_7_H_14_O_5_	178.18	80	1.2 × 10^6^
83	33.74	9h-pyrido[3,4-b]indole	244-63-3	C_11_H_8_N_2_	168.2	79.9	2.9 × 10^5^
84	15.00	5-oxotetrahydrofuran-2-carboxylic acid, ethyl ester	1129-30-2	C_7_H_10_O_4_	158.15	79.6	2.6 × 10^5^
85	14.24	benzoic acid	65-85-0	C_7_H_6_O_2_	122.12	79.1	1.6 × 10^5^
86	2.81	ethyl-1-propenyl ether	928-55-2	C_5_H_10_O	86.13	78.9	2.0 × 10^5^
87	13.15	valeric anhydride	2082-59-9	C_10_H_18_O_3_	186.25	78.9	1.6 × 10^5^
88	10.47	1,2-ethanediol, 1,2-diphenyl-	484-17-3	C_14_H_14_O_2_	214.26	78.8	1.0 × 10^5^
89	25.76	1,1,4,5,6-pentamethyl-2,3-dihydro-1h-indene	91313-26-9	C_14_H_20_	188.31	78.7	3.7 × 10^5^
90	20.63	2′,6′-dimethyl-4′-propoxyacetophenone	34443-69-1	C_13_H_18_O_2_	206.28	78.6	2.0 × 10^5^
91	3.31	crotonic anhydride	623-68-7	C_8_H_10_O_3_	154.16	78.4	4.3 × 10^4^
92	15.89	5-oxotetrahydrofuran-2-carboxylic acid, ethyl ester	001126-51-8	C_7_H_10_O_4_	158.15	78.4	1.4 × 10^6^
93	6.10	propanoic acid, 3-nitro-, methyl ester	020497-95-4	C_4_H_7_NO_4_	133.1	78.2	6.9 × 10^6^
94	2.85	acetamidoacetaldehyde	064790-08-5	C_4_H_7_NO_2_	101.1	78	2.4 × 10^5^
95	12.63	maltol	000118-71-8	C_6_H_6_O_3_	126.11	78	1.4 × 10^5^
96	20.50	naphthalene, 1,2-dihydro-4,5,7-trimethyl-	053156-11-9	C_13_H_16_	172.27	78	3.3 × 10^4^
97	7.90	2,3-butanedione monoxime	57-71-6	C_4_H_7_NO_2_	101.1	77.9	7.2 × 10^4^
98	22.66	1h-indol-3-ol, acetate	000608-08-2	C_10_H_9_NO_2_	175.18	77.9	7.1 × 10^5^
99	22.66	4-(2,6,6-trimethylcyclohexa-1,3-dienyl)but-3-en-2-one	001203-08-3	C_13_H_18_O	190.28	77.9	3.3 × 10^5^
100	8.57	2(3h)-furanone, 5-acetyldihydro-	029393-32-6	C_6_H_8_O_3_	128.13	77.8	6.1 × 10^4^
101	13.15	propylhydrazone propionaldehyde	000000-00-0	C_6_H_14_N_2_	114.19	77.8	2.4 × 10^5^
102	21.85	benzaldehyde, 2-hydroxy-6-methyl-	018362-36-2	C_8_H_8_O_2_	136.15	77.8	3.6 × 10^6^
103	26.62	homovanillic acid	000306-08-1	C_9_H_10_O_4_	182.17	77.8	7.9 × 10^5^
104	25.42	aminomaleimide	037770-94-8	C_4_H_4_N_2_O_2_	112.09	77.6	2.9 × 10^6^
105	26.14	1-(4-tert-butylphenyl)propan-2-one	081561-77-5	C_13_H_18_O	190.28	77.5	2.0 × 10^5^
106	6.70	crotonyl isothiocyanate	060034-28-8	C_5_H_5_NOS	127.16	77.4	5.0 × 10^5^
107	13.38	methane, isocyanato-	000624-83-9	C_2_H_3_NO	57.05	77.3	2.8 × 10^5^
108	12.19	cyclopropyl carbinol	002516-33-8	C_4_H_8_O	72.11	77.2	2.5 × 10^5^
109	1.75	ethyl vinyl ether	000000-00-0	C_4_H_8_O	72.11	76.8	5.9 × 10^5^
110	5.61	furan, 2-methoxy-	025414-22-6	C_5_H_6_O_2_	98.1	76.8	3.7 × 10^4^
111	9.39	n-butyl-tert-butylamine	016486-74-1	C_8_H_19_N	129.24	76.8	1.3 × 10^5^
112	28.63	4-((1e)-3-hydroxy-1-propenyl)-2-methoxyphenol	458-35-5	C_6_H_8_O_3_	128.13	76.7	8.0 × 10^5^
113	9.28	.alpha.-acetobutyrolactone	000517-23-7	C_6_H_8_O_3_	128.13	76.4	4.0 × 10^5^
114	3.08	n-propyl acetate	000109-60-4	C_5_H_10_O_2_	102.13	76.3	3.3 × 10^6^
115	19.54	1, 1, 5-trimethyl-1, 2-dihydronaphthalene	30364-33-1	C_13_H_16_	172.27	76.2	4.0 × 10^4^
116	1.47	acetic anhydride	000108-24-7	C_4_H_6_O_3_	102.09	76	1.1 × 10^5^
117	17.77	1-[(1-oxo-2-propenyl)oxy]-2,5-pyrrolidinedione	038862-24-7	C_7_H_7_NO_4_	169.14	76	8.3 × 10^5^
118	19.31	phenol, 2,6-dimethoxy-	000091-10-1	C_8_H_10_O_3_	154.16	76	1.2 × 10^5^
119	29.92	cyclopentane, 1,1,3,3-tetramethyl-	4516-69-2	C_9_H_18_	126.24	75.9	6.7 × 10^4^
120	40.07	toluene	108-88-3	C_7_H_8_	92.14	75.8	5.3 × 10^4^
121	1.99	dimethyl ether	000115-10-6	C_2_H_6_O	46.07	75.7	1.8 × 10^5^
122	20.50	naphthalene, 1,2-dihydro-4,5,7-trimethyl-	053156-11-9	C_13_H_16_	172.27	75.7	5.6 × 10^4^
123	2.63	trimethyl orthoformate	000000-00-0	C_4_H_10_O_3_	106.12	75.6	2.0 × 10^4^
124	10.72	p-dioxane, 2,5-dimethyl-3-methylene-	003984-21-2	C_7_H_12_O_2_	128.17	75.4	2.7 × 10^6^
125	18.26	triethylene glycol diacetate	149-73-5	C_10_H_18_O_6_	234.25	75.4	2.4 × 10^5^
126	31.44	methanol, [4-(1,1-dimethylethyl)phenoxy]-, acetate	054889-98-4	C_13_H_18_O_3_	222.28	75.4	2.6 × 10^5^
127	4.26	2-pyrrolidinone, 5-(cyclohexylmethyl)-	014293-08-4	C_11_H_19_NO	181.27	75.3	2.5 × 10^5^
128	6.81	2(3h)-furanone	020825-71-2	C_4_H_4_O_2_	84.07	75.2	1.3 × 10^5^
129	4.79	pyrazine, methyl-	000109-08-0	C_5_H_6_N_2_	94.11	75.1	5.9 × 10^5^
130	13.38	2-acetyl-2,3,5,6-tetrahydro-1,4-thiazine	656-42-8	C_6_H_11_NOS	145.22	75	5.1 × 10^5^

**Table 2:** AgNP synthesis with *R. stolonifer* fungus extracts after 3 and 11 days of growth for different stress times after 24 h of reaction. Note: Data that did not meet the three conditions stipulated for AgNP formation with *R. stolonifer* fungus extracts are shown in bold lettering.

Sample Code	Fungus Age, Days	Stress Time, h	Polyphenols, mg mL^−1^	Proteins, mg mL^−1^	Protein/Polyphenols Rate	Wavelength, nm
E3-2d	3	2	0.024	0.788	32.7	495
**E3-48a**	3	48	0.065	**1.476**	22.7	**452**
E3-2c	3	2	0.046	0.943	20.4	434
**E3-48d**	3	48	0.070	**1.271**	18.0	**0**
E11-48a	11	48	0.054	0.815	15.0	441
E11-96b	11	96	0.061	0.893	14.7	442
E11-24d	11	24	0.069	0.947	13.8	447
E11-846	11	46	0.035	0.414	11.8	450
E11-48c	11	48	0.073	0.854	11.7	448
E3-24d	3	24	0.074	0.863	11.6	0
**E11-2a**	11	2	0.106	**1.181**	11.2	**0**
E11-24c	11	24	0.081	0.899	11.1	451
**E3-72b**	3	72	0.117	**1.270**	10.8	**455**
E11-2c	11	2	0.087	0.906	10.4	0
E3-746	3	46	0.041	0.424	10.3	450
E11-946	11	46	0.041	0.421	10.3	450
E11-72a	11	72	0.084	0.833	9.9	0
**E3-2b**	3	2	0.109	**1.069**	9.8	**0**
E3-72a	3	72	0.087	0.856	9.8	453
E3-446	3	46	0.043	0.414	9.7	0
E11-48b	11	48	0.074	0.713	9.6	445
**E11-2d**	11	2	**0.131**	**1.243**	9.5	**0**
E3-24c	3	24	0.096	0.879	9.2	457
E3-96d	3	96	0.065	0.580	9.0	0
E3-230	3	0.5	0.047	0.410	8.6	434
E3-530	3	0.5	0.048	0.414	8.6	437
E3-727	3	27	0.048	0.410	8.5	451
E3-427	3	27	0.049	0.412	8.4	446
E11-24a	11	24	0.093	0.769	8.3	442
E11-827	11	27	0.051	0.418	8.2	437
E3-720	3	20	0.051	0.412	8.1	443
E3-546	3	46	0.053	0.422	8.0	450
E11-830	11	0.5	0.052	0.412	8.0	446
E11-930	11	0.5	0.052	0.412	8.0	443
E3-730	3	0.5	0.051	0.399	7.9	429
E11-1027	11	27	0.054	0.423	7.8	450
E11-2b	11	2	0.098	0.757	7.7	0
E3-724	3	24	0.054	0.417	7.7	449
E11-72b	11	72	0.114	0.868	7.6	450
**E3-48c**	3	48	**0.181**	**1.375**	7.6	**0**
E11-96d	11	96	0.101	0.754	7.4	441
E11-72d	11	72	0.097	0.718	7.4	456
E3-627	3	27	0.057	0.418	7.4	446
E11-820	11	20	0.055	0.405	7.3	435
E3-246	3	46	0.057	0.417	7.3	0
E3-130	3	0.5	0.058	0.412	7.1	434
E3-527	3	27	0.060	0.423	7.0	470
**E3-24b**	3	24	**0.226**	**1.567**	6.9	**0**
**E3-72d**	3	72	**0.232**	**1.608**	6.9	**0**
E11-1046	11	46	0.061	0.419	6.9	450
E3-420	3	20	0.059	0.403	6.8	446
E3-327	3	27	0.062	0.418	6.7	0
E3-646	3	46	0.064	0.427	6.7	0
**E3-96a**	3	96	**0.172**	**1.133**	6.6	**0**
E11-920	11	20	0.061	0.404	6.6	437
**E3-48b**	3	48	**0.167**	**1.094**	6.6	**0**
E11-927	11	27	0.061	0.400	6.5	450
E11-1020	11	20	0.063	0.409	6.5	434
E11-24b	11	24	0.113	0.728	6.4	443
E11-96a	11	96	0.108	0.688	6.4	444
E3-430	3	0.5	0.064	0.403	6.3	429
E3-220	3	20	0.065	0.406	6.2	446
E3-330	3	0.5	0.065	0.402	6.2	425
E11-72c	11	72	0.109	0.672	6.2	440
E11-48d	11	48	0.109	0.660	6.1	445
E3-630	3	0.5	0.068	0.407	6.0	437
E11-1024	11	24	0.074	0.432	5.9	435
**E3-24a**	3	24	**0.150**	0.863	5.8	**454**
E3-624	3	24	0.074	0.418	5.6	0
E3-224	3	24	0.077	0.419	5.4	451
E11-1030	11	0.5	0.074	0.402	5.4	446
**E11-96c**	11	96	**0.134**	0.720	5.4	**442**
E3-320	3	20	0.076	0.406	5.3	444
E3-520	3	20	0.077	0.411	5.3	451
E11-924	11	24	0.082	0.427	5.2	440
E3-524	3	24	0.083	0.429	5.1	459
E11-824	11	24	0.084	0.414	4.9	435
E3-424	3	24	0.083	0.406	4.9	446
E3-124	3	24	0.084	0.411	4.9	0
E3-324	3	24	0.086	0.412	4.8	464
E3-227	3	27	0.090	0.423	4.7	451
E3-146	3	46	0.090	0.412	4.6	0
**E3-72c**	3	72	**0.259**	**1.048**	**4.0**	**0**
**E3-120**	3	20	0.100	0.402	**4.0**	**0**
**E3-620**	3	20	0.104	0.410	**4.0**	**443**
**E3-96c**	3	96	**0.160**	0.627	**3.9**	**0**
**E3-346**	3	46	0.105	0.405	**3.9**	**0**
**E3-2a**	3	2	**0.133**	0.512	**3.8**	**0**
**E3-96b**	3	96	**0.190**	0.552	**2.9**	**0**
**E3-127**	3	27	**0.155**	0.426	**2.7**	**0**

**Table 3:** Sizes of the biosynthesized AgNPs observed by HRTEM microscopy.

Range	Sample Code	Polyphenols, mg mL^−1^	Proteins, mg mL^−1^	Rate	Observed Size, nm
1	S3-524	0.083	0.429	5	21 ± 4
2	S3-24a	0.150	0.863	6	31 ± 27
	S11-24c	0.081	0.899	11	71 ± 15
	S11-48c	0.073	0.854	12	50 ± 25
3	S3-320	0.076	0.406	5	19 ±9
	S11-827	0.051	0.418	8	25 ± 10
4	S3-430	0.064	0.403	6	20 ± 4
	S11-824	0.084	0.414	5	30 ± 8

## Data Availability

The original contributions presented in this study are included in the article/Supplementary Material. Further inquiries can be directed to the corresponding authors.

## Supplementary Materials

The following supporting information can be downloaded at: https://www.mdpi.com/article/10.3390/molecules31173103/s1, Figure S1: Biomass production kinetics of the fungus *R. stolonifer* grown on PDA; Figure S2: UV spectra of the different extracts of the fungus *R. stolonifer* (a) for 3 days of growth and (b) for 11 days growth; Figure S3: FT-IR spectra of different *R. stolonifer* fungus extracts after 3 days of growth; Figure S4: Representative GC-MS total-ion chromatogram for *R. stolonifer* extract. Annotations of representative metabolites are displayed; Figure S5: Comparison of experimental and library mass spectra for phenolic compounds. Head-to-tail comparison of experimental mass fragmentation against NIST-MS library for (a) phenol, (b) 1,2-benzenediol, (c) p-cresol, (d) p-hydroxybenzyl alcohol, (e) 4-vinylguaiacol, (f) benzaldehyde, 2-hydroxy-6-methyl- Identifications are based on a match factor ≥ 80, as detailed in Table 1; Figure S6: Comparison of experimental and library mass spectra for methoxylated phenolic compounds. Head to tail comparison of experimental mass fragmentation against NIST-MS library for (a) o-methoxyphenol, (b) 2-(1-propenyl)-6methoxyphenol, (c) 2-(1-propenyl)-6-methoxyphenol, (d) homovanillic acid, (e) phenol, 2,6-dimethoxy-, (f) phenol, 2,6-dimethoxy-Identifications are based on a match factor ≥ 80 as detailed in Table 1; Figure S7: Comparison of experimental and library mass spectra for methoxylated phenolic compounds. Head-to-tail comparison of experimental mass fragmentation against NIST-MS library for (a) furfural, (b) 2,4-dihydroxy-2,5-dimethyl-3(2H)-furan-3-one, (c) 5-hydroxymethylfurfural, (d) 5-methylfurfural, (e) 2-methylpyrazine, (f) uracil. Identifications are based on a match factor ≥ 80 as detailed in Table 1; Figure S8: UV-vis spectra range of the synthesized AgNPs; Range 1: 435–425 nm; Figure S9. UV-vis spectra range of the synthesized AgNPs; Range 2: 455–446 nm. Figure S10. UV-vis spectra range of the synthesized AgNPs; Range 3: 445–437 nm. Figure S11. UV-vis spectra range of the synthesized AgNPs; Range 4: 435–425 nm. Figure S12: FT-IR spectra of *R. stolonifer* fungus extracts and biogenically synthesized AgNPs; Figure S13: HRTEM micrographs of the AgNPs in (a) Range 1, (b) Range 2, (c) Range 3, and (d) Range 4; Figure S14: Results of disinfection tests of AgNPs synthesized with R. stolonifer fungus extracts (a) *E. coli* y (b) *S. aureus*; Table S1: Elemental analysis of AgNPs biosynthesized with fungus extracts.

## Abbreviations

The following abbreviations are used in this manuscript:

AgNPs	Silver nanoparticles
NPs	Nanoparticles
PDA	Potato dextrose agar
BSA	Bovine serum albumin
SPR	Surface plasmon resonance
FT-IR	Fourier Transform Infrared Spectroscopy
GC-MS	Gas chromatography–mass spectrometry
EI	Electron ionization
NIST	National Institute of Standards and Technology
UV-Vis	Ultraviolet-visible
TAR	Total reflection attenuation technique
EDS	Energy-dispersive X-ray spectroscopy
TEM	Transmission electron microscopy
MIC	Minimum Inhibitory Concentration
E3	The fungus extracts from 3 days of growth
E11	The fungus extracts from 11 days of growth
HSPs	Heat shock proteins
