# Supplementary material for: Fungal-Based Biogenic Silver Nanoparticles: Synthesis, Mathematical Modeling and Application
Source: Molecules. 2026 Sep 4;31(17):3103. doi: 10.3390/molecules31173103 (PMC13566992; doi:10.3390/molecules31173103)
Supplement: Supplementary file 1 [file molecules-31-03103-s001.zip › molecules-4440473-supplementary.pdf]

Supporting information

Fungal-Based Biogenic Silver Nanoparticles: Synthesis, Mathematical Modeling and Application

Alejandra Ortiz-Tirado [1], Erick R. Bandala [2], Victor H. Robledo-Zacarias [3], Alain S. Conejo-Davila [4,*], Alejandro Vega-Rios [5], James McGree [6], Luis Fernando Mejia-Diaz [7], Ashantha Goonetilleke [8], Martín Pacheco-Álvarez [9,10] and Oscar M. Rodriguez-Narvaez [1,*]

[1] Direction of Applied Research and Development, Center for Applied Innovation in Competitive Technologies, Omega 201, León 37545, Guanajuato, Mexico; nortiz.picyt@ciatec.mx
[2] NantBioRenewables LLC, 100 Seapoint Blv. Bldg 5, Savannah, GA 31404, USA; erick.bandala@nantrenewables.com
[3] Technological Solutions Department, Center for Applied Innovation in Competitive Technologies, Omega 201, León 37545, Guanajuato, Mexico; vrobledo@ciatec.mx
[4] MicroPrime S RL de CV, El Colorado, Santiago de Querétaro 76246, Querétaro, Mexico
[5] Centro de Investigación en Materiales Avanzados, S.C. (CIMAV), Miguel de Cervantes No. 120, Chihuahua 31136, Chihuahua, Mexico; alejandro.vega@cimav.edu.mx
[6] School of Mathematical Sciences, Queensland University of Technology, GPO Box 2344, Brisbane, QLD 4001, Australia; james.mcgree@qut.edu.au
[7] Transdisciplinary Institute of Research and Services (ITRANS), University of Guadalajara, Zapopan 45150, Jalisco, Mexico; mejia.fer93@gmail.com
[8] Department of Civil Engineering, Birla Institute of Technology and Science, Pilani Campus, Vidya Vihar, Pilani 333031, Rajasthan, India; ashanthag@pm.me
[9] Departamento de Química-DCNE, Universidad de Guanajuato, Cerro de la Venada s/n, Pueblito de Rocha, Guanajuato 36040, Guanajuato, Mexico; moa.pachecoalvarez@ugto.mx
[10] Tecnológico Nacional de México, Instituto Tecnológico Superior de Guanajuato, Carretera Estatal Guanajuato-Puentecillas Km. 10.5, Guanajuato 36262, Guanajuato, Mexico
* Correspondence: conejo_salvador@hotmail.com (A.S.C.-D.); orodriguez@ciatec.mx (O.M.R.-N.); Tel.: +52-614-255-3815 (A.S.C.-D.); +52-311-121-52-36 (O.M.R.-N.)

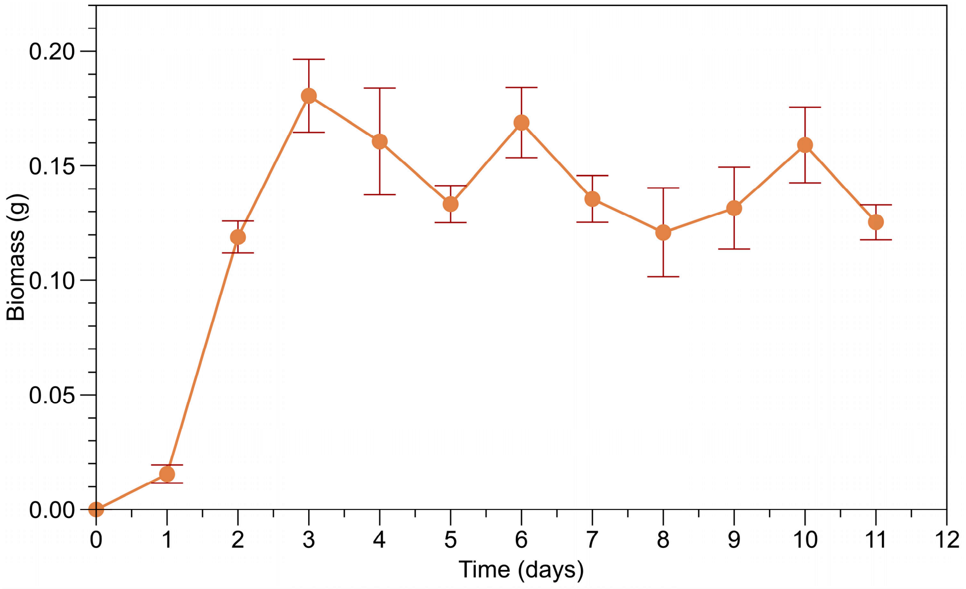

Figure S1. Biomass production kinetics of the fungus *R. stolonifer* grown on PDA

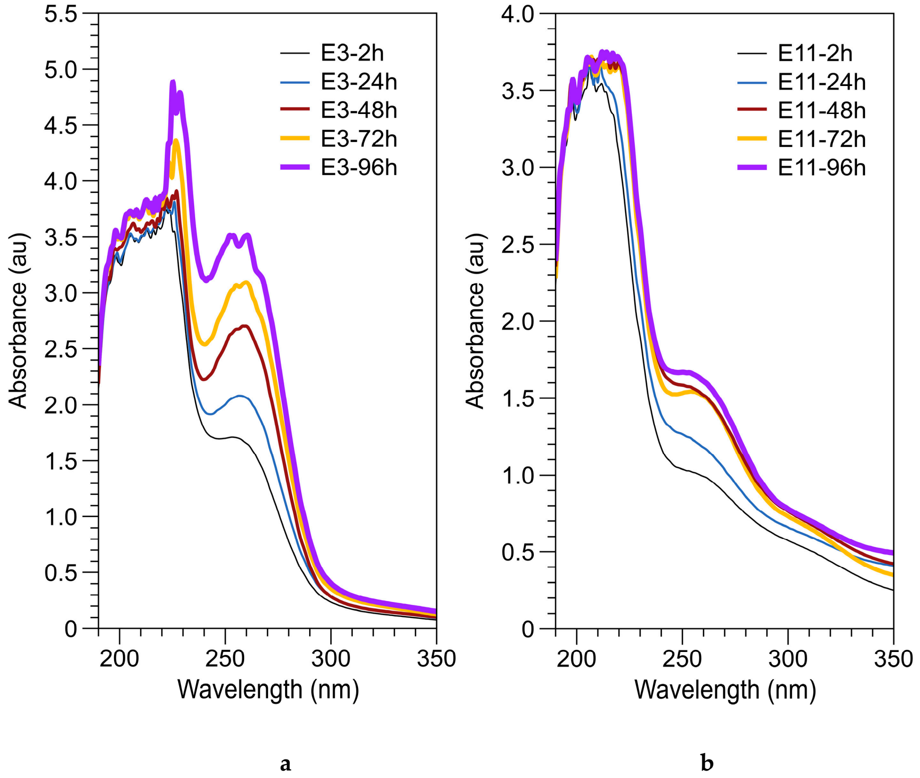

Figure S2. UV spectra of the different extracts of the fungus *R. stolonifer* a) for 3 days of growth and b) for 11 days growth.

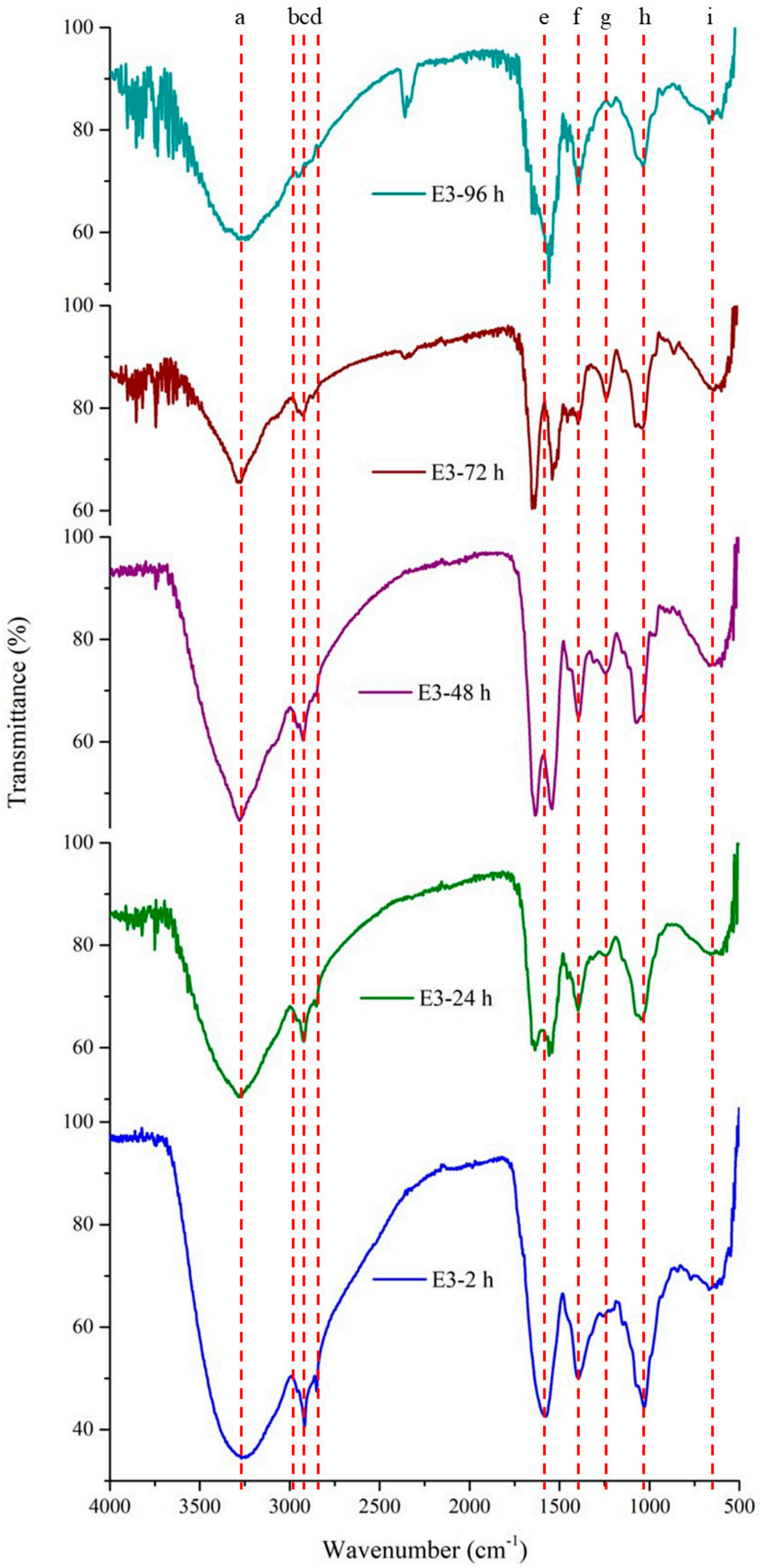

Figure S3. FT-IR spectra of different *R. stolonifer* fungus extracts after 3 days of growth.

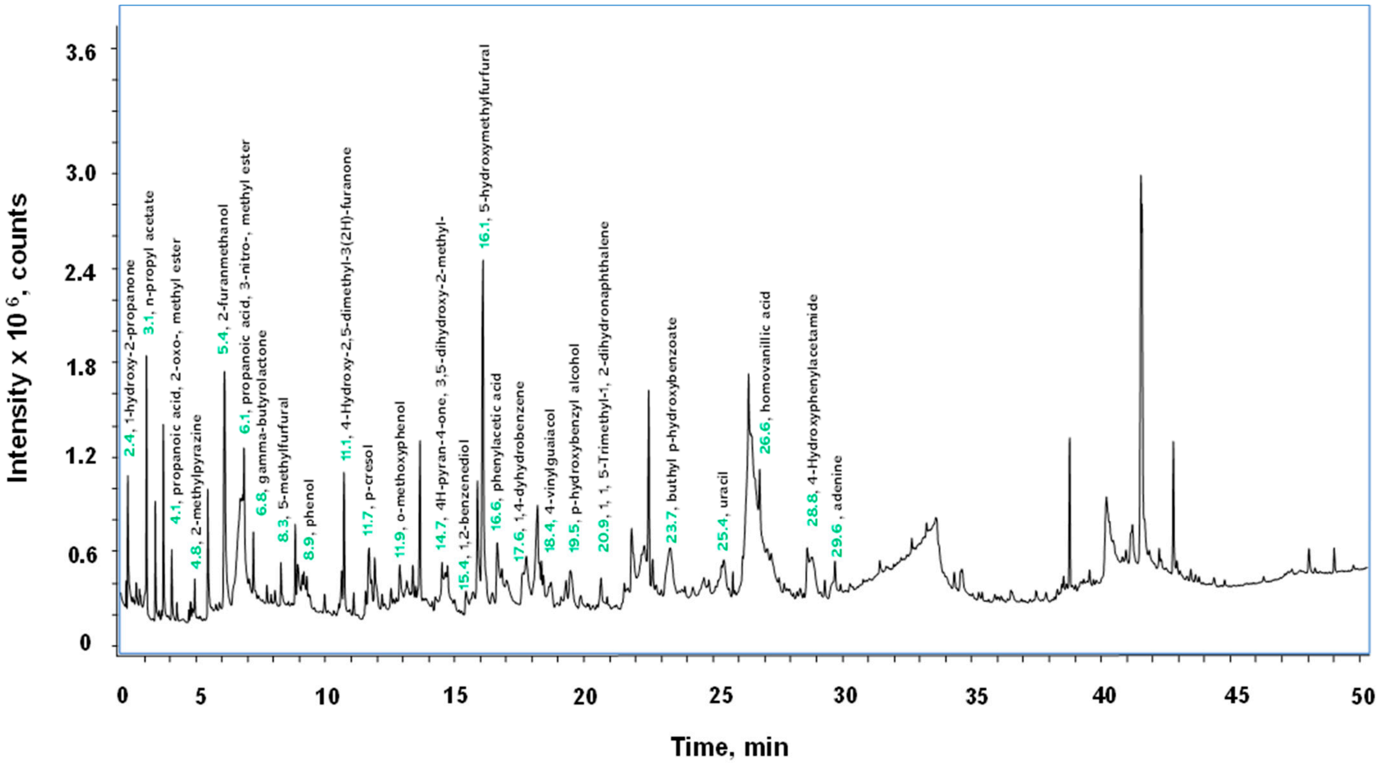

Figure S4. Representative GC-MS total-ion chromatogram for *R. stolonifer* extract. Annotations of representative metabolites are displayed.

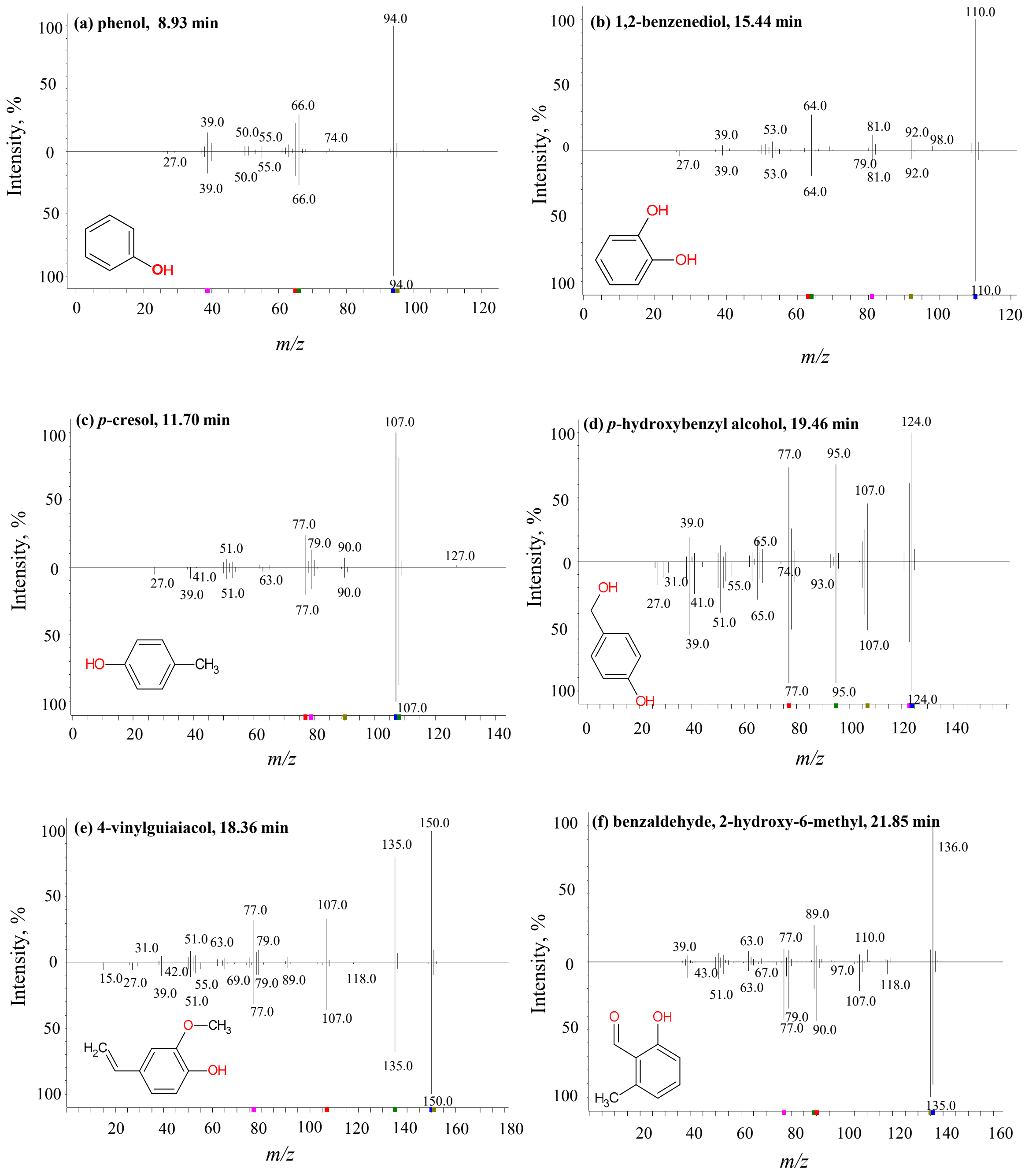

Figure S5. Comparison of experimental and library mass spectra for phenolic compounds. Head-to-tail comparison of experimental mass fragmentation against NIST-MS library for (a) phenol, (b) 1,2-benzenediol, (c) p-cresol, (d) p-hydroxybenzyl alcohol, (e) 4-vinylguaiacol, (f) benzaldehyde, 2-hydroxy-6-methyl- Identifications are based on a match factor ≥ 80 as detailed in Table 1.

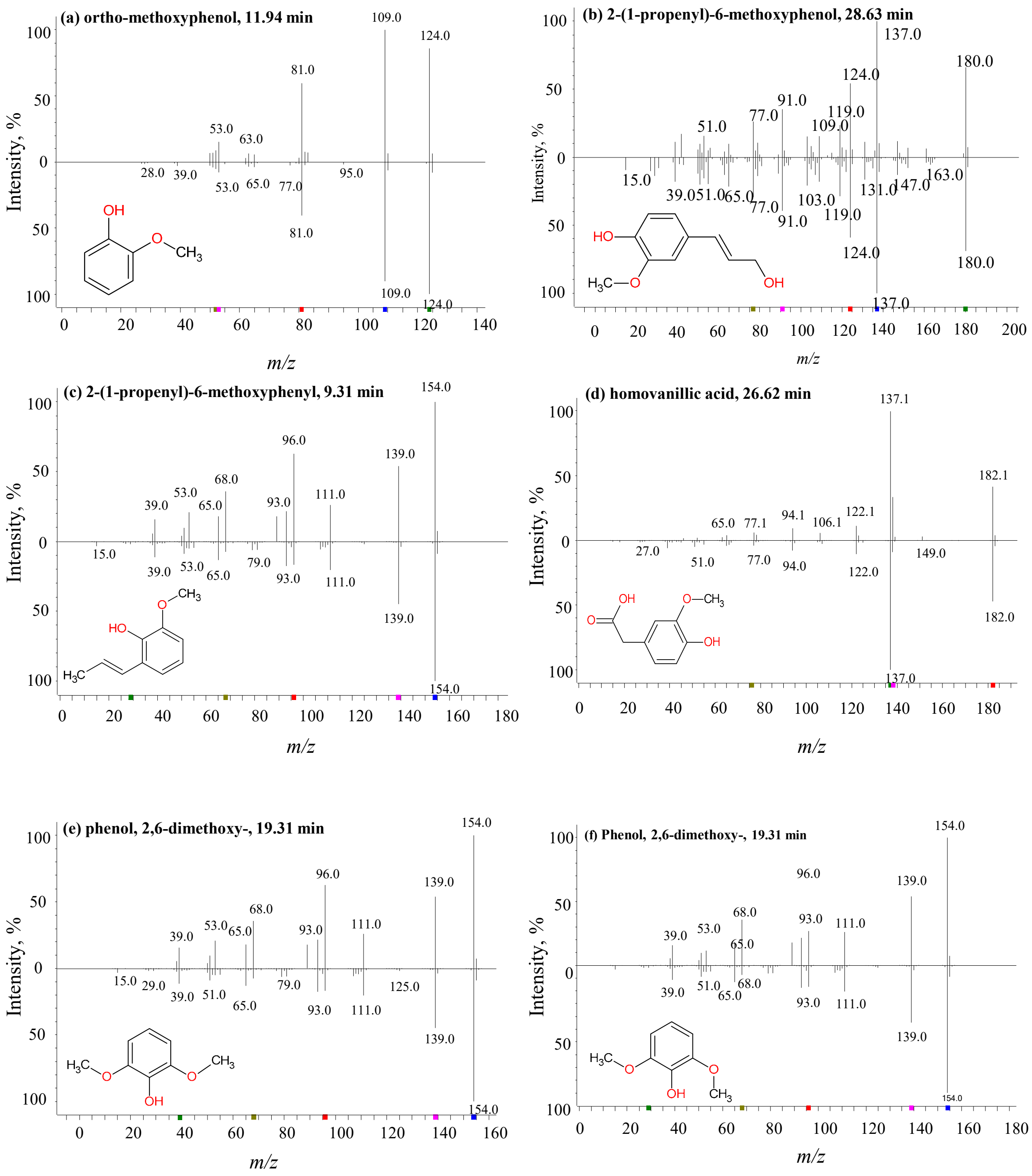

Figure S6. Comparison of experimental and library mass spectra for methoxylated phenolic compounds. Head to tail comparison of experimental mass fragmentation against NIST-MS library for (a) o-methoxyphenol, (b) 2-(1-propenyl)-6methoxyphenol, (c) 2-(1-propenyl)-6-methoxyphenol, (d) homovanillic acid, (e) phenol, 2,6-dimethoxy-, (f) phenol, 2,6-dimethoxy-Identifications are based on a match factor ≥ 80 as detailed in Table 1.

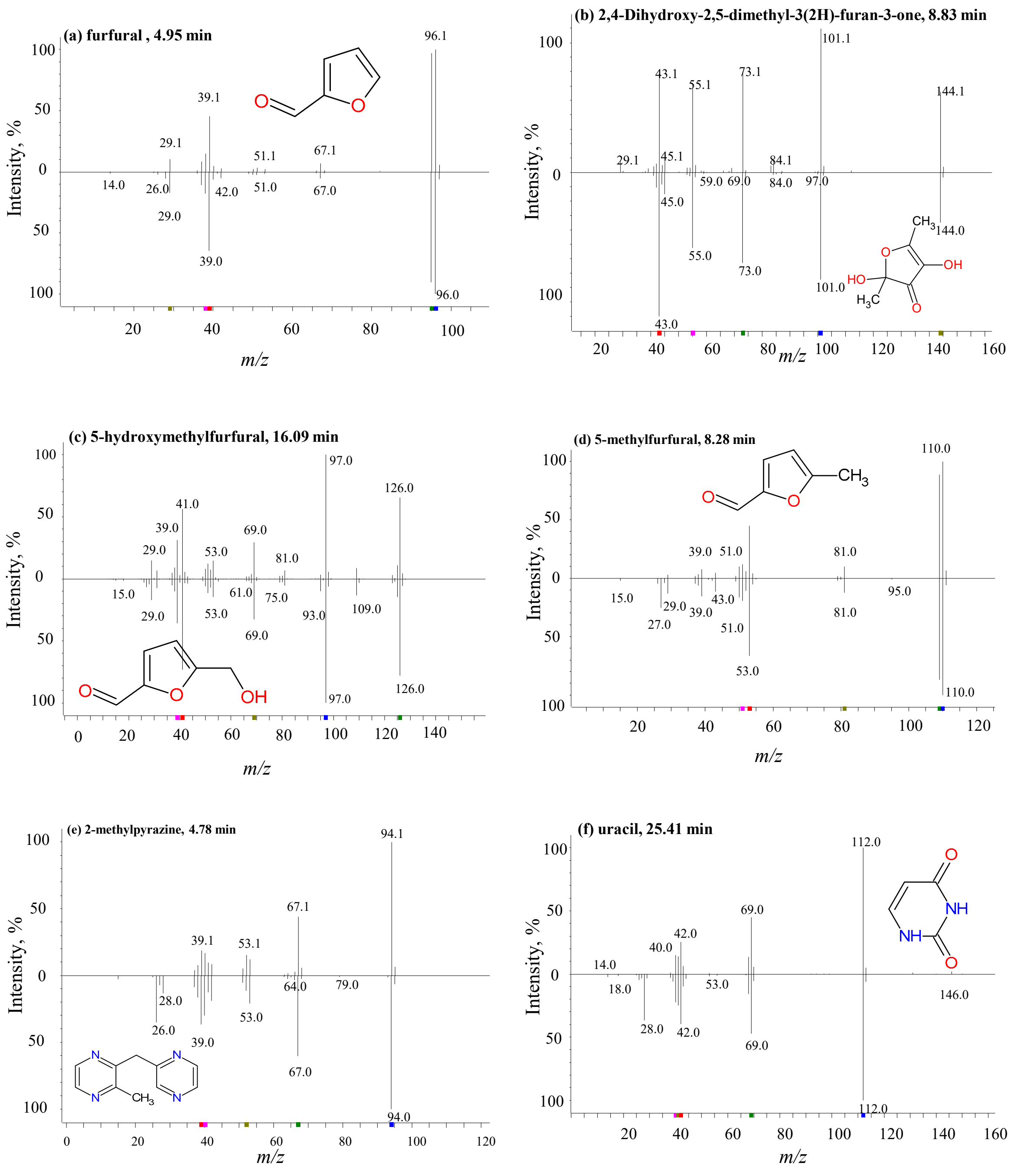

Figure S7. Comparison of experimental and library mass spectra for methoxylated phenolic compounds. Head-to-tail comparison of experimental mass fragmentation against NIST-MS library for (a) furfural, (b) 2,4-dihydroxy-2,5-dimethyl-3(2H)-furan-3-one, (c) 5-hydroxymethylfurfural, (d) 5-methylfurfural, (e) 2-methylpyrazine, (f) uracil. Identifications are based on a match factor ≥ 80 as detailed in Table 1.

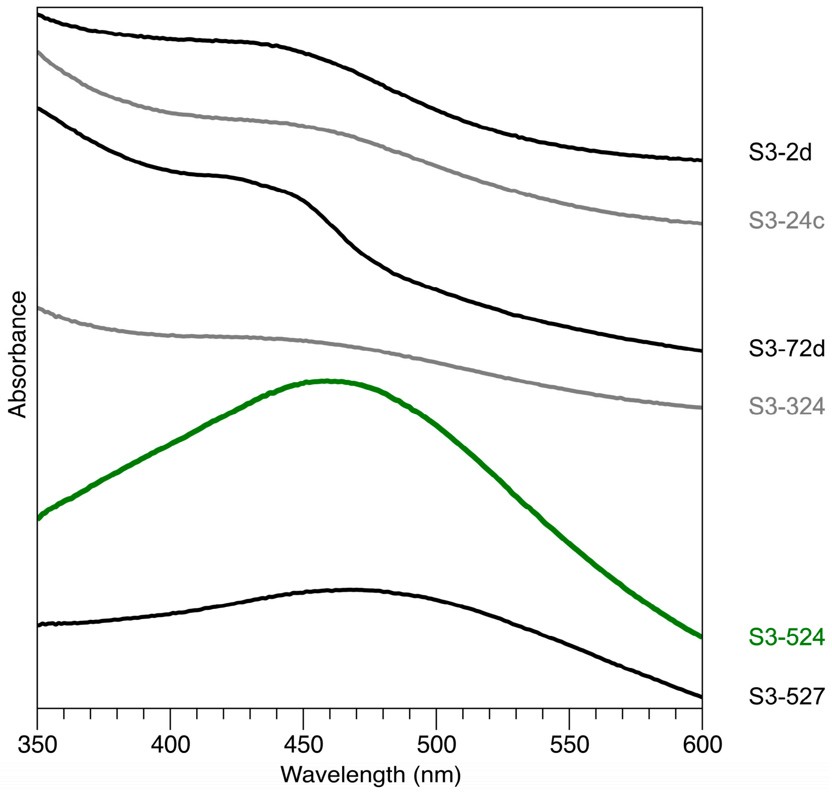

Figure S8. UV-vis spectra range of the synthesized AgNPs; Range 1: 435-425 nm.

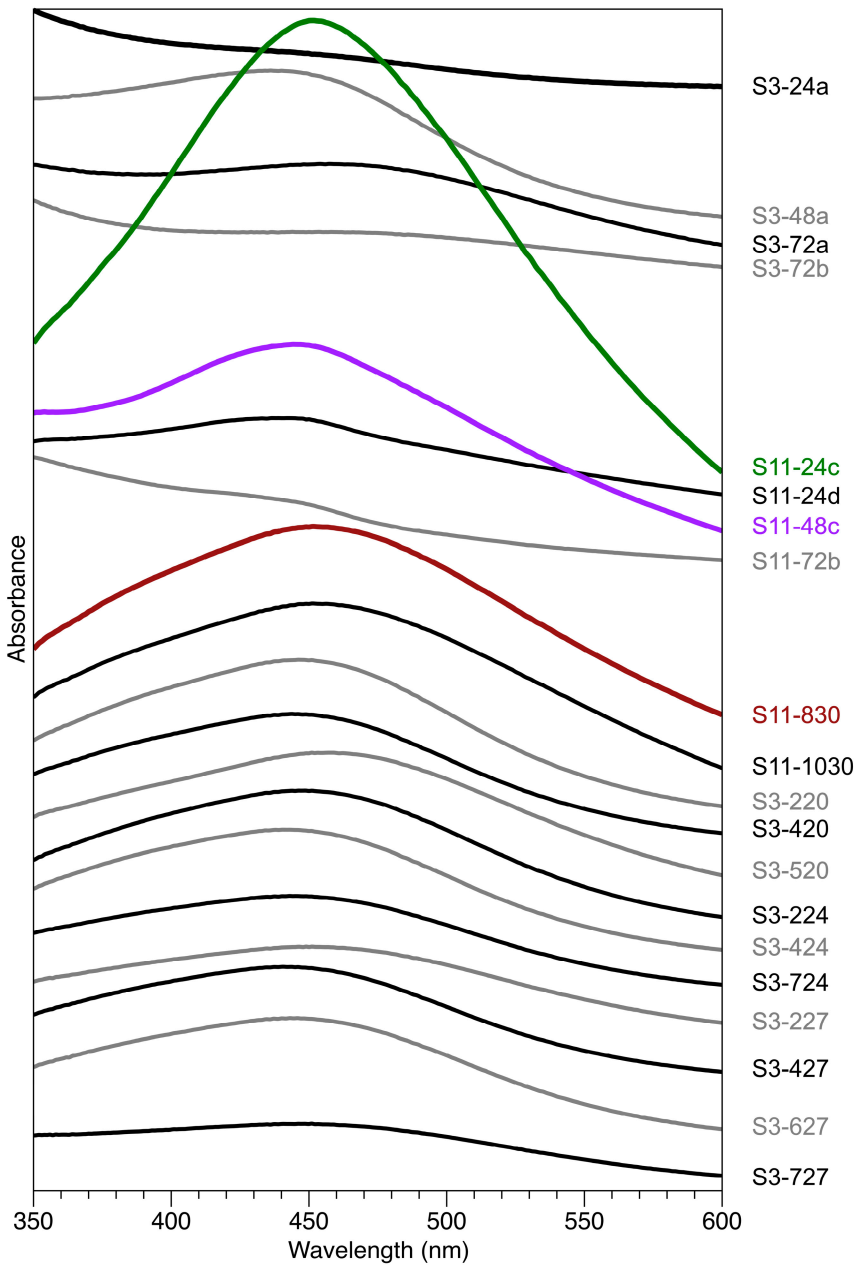

Figure S9. UV-vis spectra range of the synthesized AgNPs; Range 2: 455-446 nm.

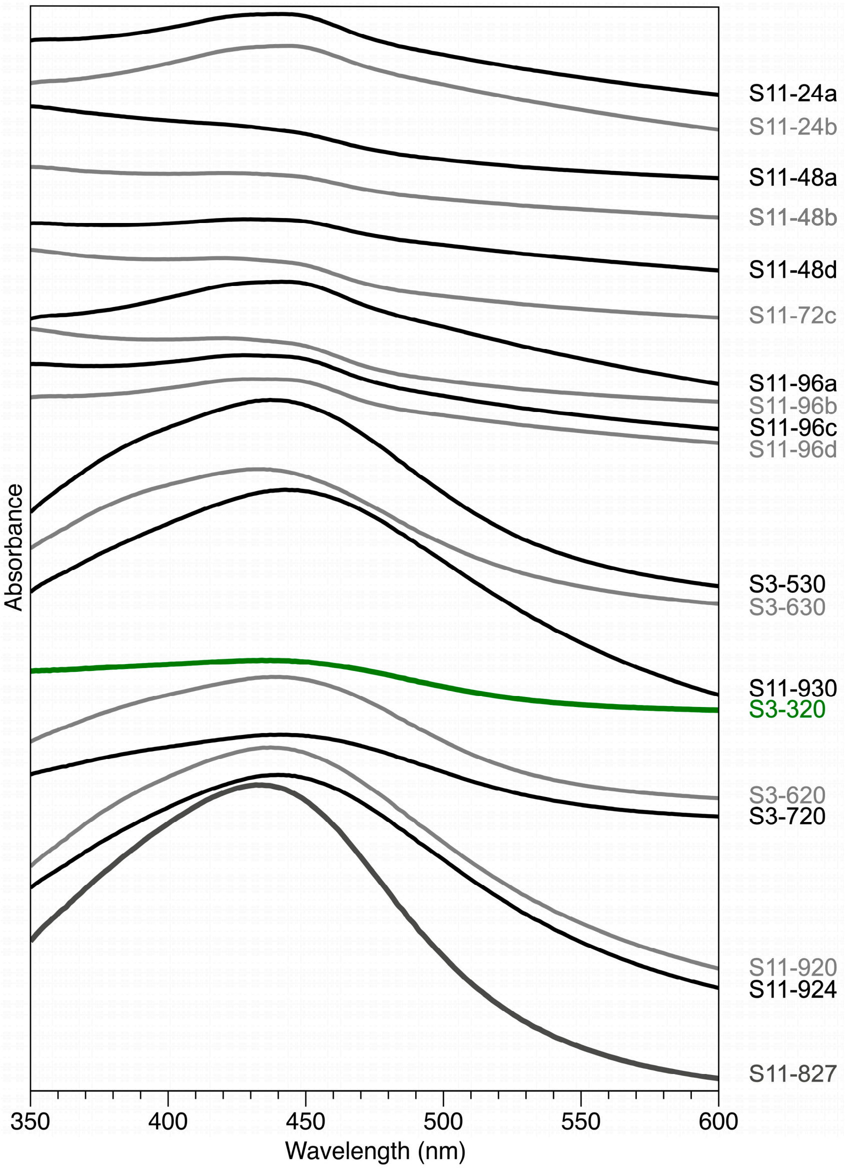

Figure S10. UV-vis spectra range of the synthesized AgNPs; Range 3: 445-437 nm.

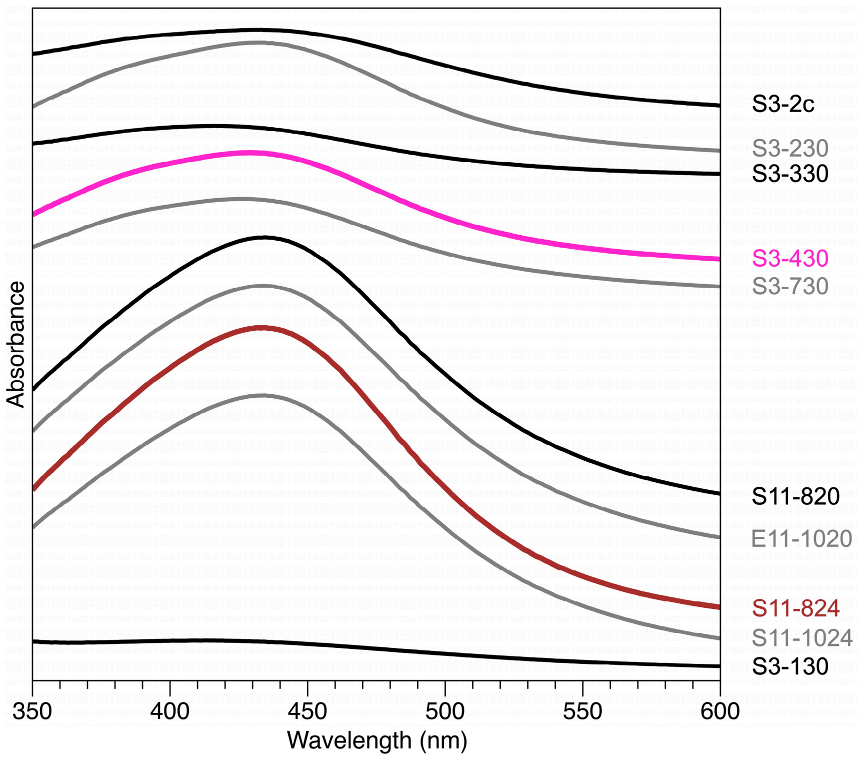

Figure S11. UV-vis spectra range of the synthesized AgNPs; Range 4: 435-425 nm.

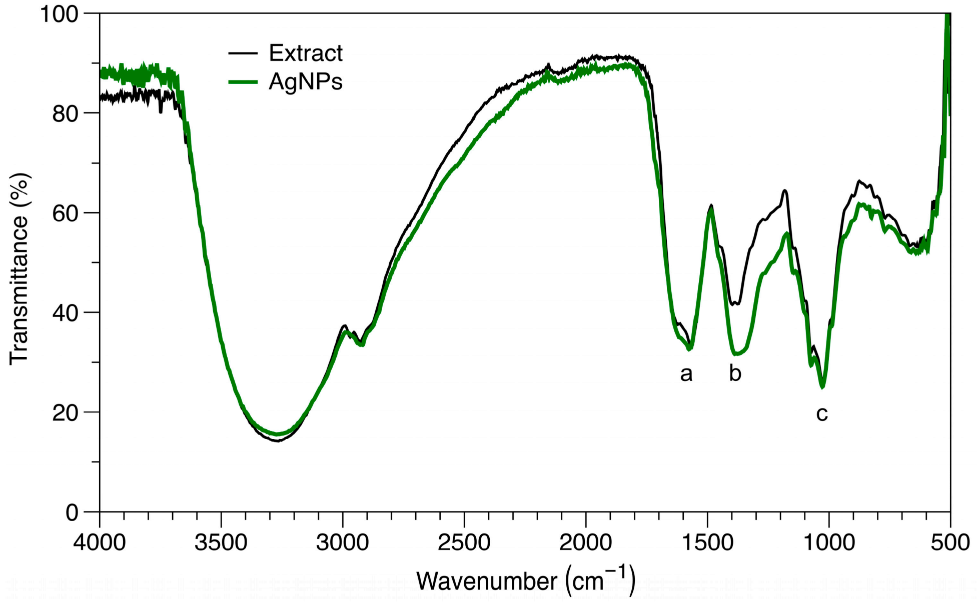

Figure S12. FT-IR spectra of R. stolonifer fungus extracts and biogenically synthesized AgNPs.

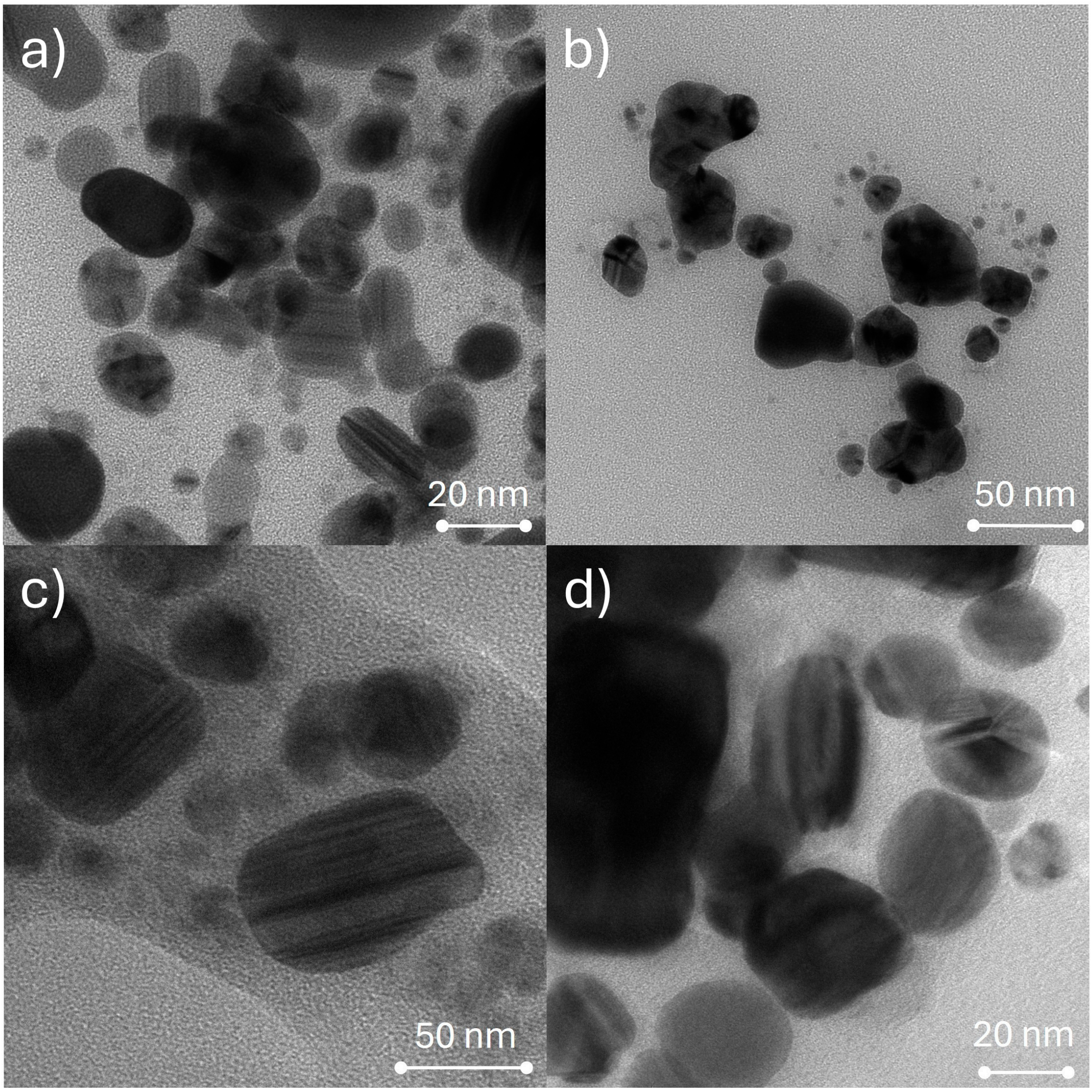

Figure S13. HRTEM micrographs of the AgNPs in a) Range 1, b) Range 2, c) Range 3, and d) Range 4.

Table S1. Elemental analysis of AgNPs biosynthesized with fungus extracts.

Sample code/ Range	Polyphenols	Proteins	Rate	Elemental Analysis		
				%C	%Ag	%O
**S11-24c/ 1**	0.081	0.899	11	15	60	5
**S11-48d/ 3**	0.109	0.660	6	80	7	10
**S3-72a/ 2**	0.087	0.856	10	4	95	1
**S11-96d/ 3**	0.101	0.754	7	60	28	8

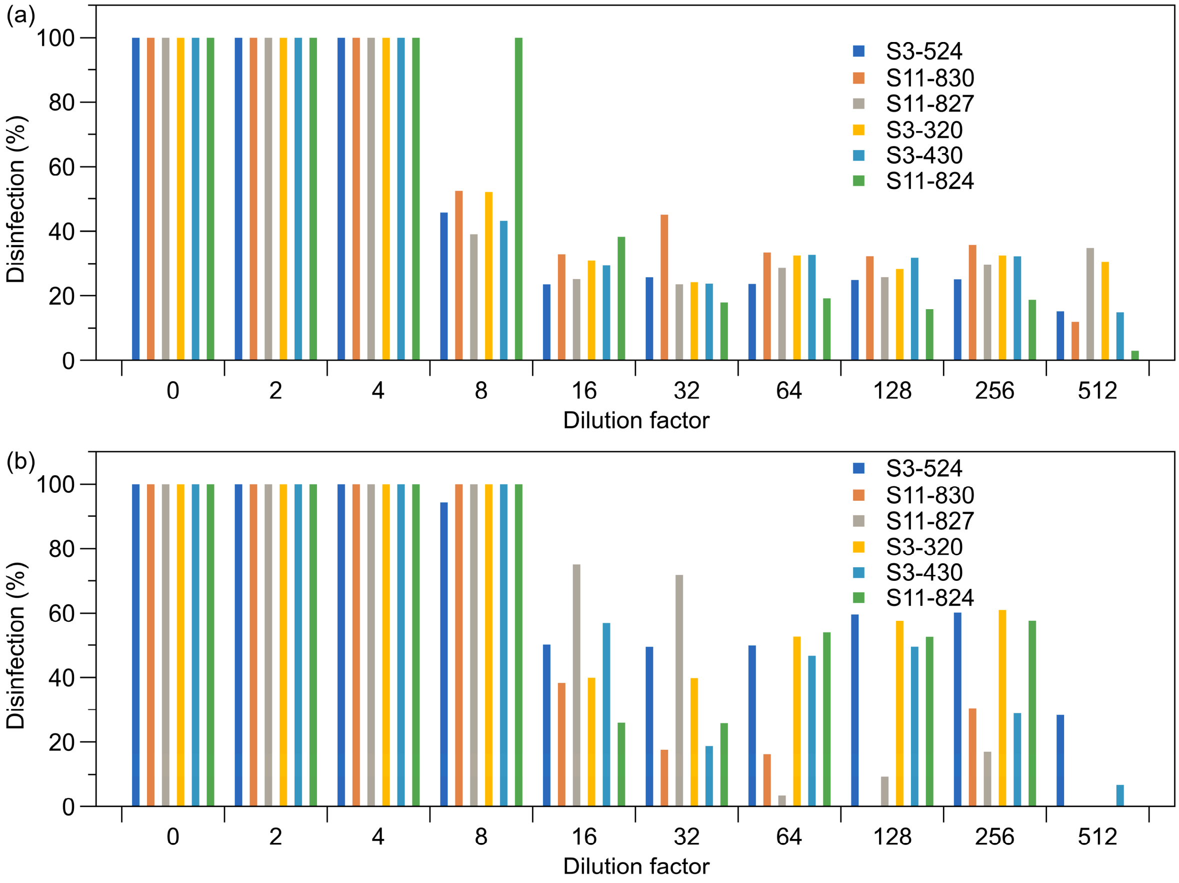

Figure S14. Results of disinfection tests of AgNPs synthesized with *R. stolonifer* fungus extracts a) *E. coli* y b*) S. aureus*.
